# Efficacy of Antibiotic Combinations against Multidrug-Resistant Pseudomonas aeruginosa in Automated Time-Lapse Microscopy and Static Time-Kill Experiments

**DOI:** 10.1128/AAC.02111-19

**Published:** 2020-05-21

**Authors:** Anna Olsson, Pikkei Wistrand-Yuen, Elisabet I. Nielsen, Lena E. Friberg, Linus Sandegren, Pernilla Lagerbäck, Thomas Tängdén

**Affiliations:** a Department of Medical Sciences, Uppsala University, Uppsala, Sweden; b Department of Pharmaceutical Biosciences, Uppsala University, Uppsala, Sweden; c Department of Medical Biochemistry and Microbiology, Uppsala University, Uppsala, Sweden

**Keywords:** carbapenem resistance, Gram-negative bacteria, combination therapy, synergy, polymyxins

## Abstract

Antibiotic combination therapy is used for severe infections caused by multidrug-resistant (MDR) Gram-negative bacteria, yet data regarding which combinations are most effective are lacking. This study aimed to evaluate the *in vitro* efficacy of polymyxin B in combination with 13 other antibiotics against four clinical strains of MDR Pseudomonas aeruginosa. We evaluated the interactions of polymyxin B in combination with amikacin, aztreonam, cefepime, chloramphenicol, ciprofloxacin, fosfomycin, linezolid, meropenem, minocycline, rifampin, temocillin, thiamphenicol, or trimethoprim by automated time-lapse microscopy using predefined cutoff values indicating inhibition of growth (≤10^6^ CFU/ml) at 24 h. Promising combinations were subsequently evaluated in static time-kill experiments. All strains were intermediate or resistant to polymyxin B, antipseudomonal β-lactams, ciprofloxacin, and amikacin. Genes encoding β-lactamases (e.g., *bla*_PAO_ and *bla*_OXA-50_) and mutations associated with permeability and efflux were detected in all strains. In the time-lapse microscopy experiments, positive interactions were found with 39 of 52 antibiotic combination/bacterial strain setups. Enhanced activity was found against all four strains with polymyxin B used in combination with aztreonam, cefepime, fosfomycin, minocycline, thiamphenicol, and trimethoprim. Time-kill experiments showed additive or synergistic activity with 27 of the 39 tested polymyxin B combinations, most frequently with aztreonam, cefepime, and meropenem. Positive interactions were frequently found with the tested combinations, against strains that harbored several resistance mechanisms to the single drugs, and with antibiotics that are normally not active against P. aeruginosa. Further study is needed to explore the clinical utility of these combinations.

## Abstract (precis)

Antibiotic combination therapy is used for severe infections caused by multidrug-resistant (MDR) Gram-negative bacteria, yet data regarding which combinations are most effective are lacking. This study aimed to evaluate the *in vitro* efficacy of polymyxin B in combination with 13 other antibiotics against four clinical strains of MDR Pseudomonas aeruginosa. We evaluated the interactions of polymyxin B in combination with amikacin, aztreonam, cefepime, chloramphenicol, ciprofloxacin, fosfomycin, linezolid, meropenem, minocycline, rifampin, temocillin, thiamphenicol, or trimethoprim by automated time-lapse microscopy using predefined cutoff values indicating inhibition of growth (≤10^6^ CFU/ml) at 24 h.

## INTRODUCTION

The emergence and spread of multidrug-resistant (MDR) Gram-negative bacteria are of great clinical concern (1). Mechanisms of resistance in these bacteria include impermeability, efflux, β-lactamase production, and target alterations (2–5). In cases where effective options for monotherapy are lacking, combination therapy including old antibiotics (e.g., the polymyxins) is employed to improve clinical outcome in severely ill patients (6). Polymyxin E (colistin) and polymyxin B have similar chemical structures and antibacterial activities but different pharmacokinetics, e.g., with regard to renal elimination (7). The polymyxins target the lipid A moiety of the membrane lipopolysaccharide (LPS) in Gram-negative bacteria, resulting in cell wall destruction (8). The membrane-disrupting feature of the polymyxins can also increase the intracellular concentrations of other antibiotics (9).

Because clinical evidence on antibiotic combination therapy for MDR Pseudomonas aeruginosa is lacking and difficult to assemble (10), *in vitro* data are important to identify promising regimens and provide a better understanding of the mechanisms of synergistic interaction. Time-kill experiments and checkerboard assays have demonstrated synergy with combinations of polymyxins, carbapenems, and other antibiotics against P. aeruginosa (11, 12). Automated time-lapse microscopy (oCelloScope; BioSense Solutions ApS, Farum, Denmark) has been proposed to accelerate the screening of combinations. Previous studies showed high agreement in results between the novel method and time-kill data in combination experiments against Escherichia coli and Klebsiella pneumoniae (33, 35).

In the present study, we screened the antibacterial effects of polymyxin B and 13 other antibiotics, alone and in combination, against four clinical strains of MDR P. aeruginosa using automated time-lapse microscopy. Regimens that showed positive interactions after 24 h were further evaluated in static time-kill experiments. The strains were genetically characterized using whole-genome sequencing to assess the presence of resistance genes, potential mechanisms of synergy, and genotype-phenotype associations.

(The results of this study were in part presented at the 27th European Congress of Clinical Microbiology and Infectious Diseases [ECCMID] in Vienna, Austria, and at the 28th ECCMID in Madrid, Spain.)

## RESULTS

### Antibiotic susceptibility.

All strains were multidrug resistant, displaying nonsusceptibility to antipseudomonal β-lactam antibiotics, amikacin, and ciprofloxacin according to EUCAST definitions (13), and intermediate to polymyxin B (MICs, ≤1 mg/liter), according to CLSI clinical breakpoints (Table 1) (15). EUCAST clinical breakpoints are not available for polymyxin B or the remaining antibiotics used in the study, which are normally not considered treatment options for P. aeruginosa infections.

**TABLE 1:** MIC values and classification of antibiotic susceptibility according to EUCAST clinical breakpoints v 9.0^*a*^

Antibiotic class	Antibiotic	MIC (mg/liter)			
		ARU617	ARU620	ARU622	ARU623
Polymyxins	PMB	1 (I)	0.5 (I)	0.5 (I)	0.5 (I)
β-Lactams	ATM	256 (R)	16 (S)	32 (R)	16 (S)
	FEP	32 (R)	8 (S)	16 (R)	16 (R)
	MEM	2 (S)	32 (R)	32 (R)	16 (R)
	TMC	>1,024 (NA)	>1,024 (NA)	>1,024 (NA)	>1,024 (NA)
Quinolones	CIP	2 (R)	8 (R)	16 (R)	1 (R)
Aminoglycosides	AMK	16 (I)	16 (I)	32 (R)	32 (R)
Tetracyclines	MIN	>256 (NA)	>256 (NA)	>256 (NA)	16 (NA)
Oxazolidinones	LIN	>256 (NA)	>256 (NA)	>256 (NA)	>256 (NA)
Miscellaneous	FOF	64 (NA)	128 (NA)	128 (NA)	128 (NA)
	CHL	>256 (NA)	256 (NA)	>256 (NA)	256 (NA)
	THI	>128 (NA)	64 (NA)	>128 (NA)	32 (NA)
	TMP	>32 (NA)	>32 (NA)	>32 (NA)	>32 (NA)
	RIF	32 (NA)	>32 (NA)	>32 (NA)	16 (NA)

a For polymyxin B, CLSI breakpoints (15) were applied due to the absence of EUCAST clinical breakpoints. Abbreviations: S, susceptible; I, intermediate; R, resistant; NA, not available; AMK, amikacin; ATM, aztreonam; FEP, cefepime; CHL, chloramphenicol; CIP, ciprofloxacin; FOF, fosfomycin; LIN, linezolid; MEM, meropenem; MIN, minocycline; PMB, polymyxin B; RIF, rifampin; TMC, temocillin; THI, thiamphenicol; TMP, trimethoprim.

### Genetic characterization.

All strains harbored genes encoding β-lactamases (e.g., *bla*_PAO_ and *bla*_OXA-50_) and enzymes that inactivate fosfomycin (*fosA*), chloramphenicol (*catB7*), and aminoglycosides [*aph(3′)-Iib*] (Table 2). Additional aminoglycoside-modifying enzymes were detected in ARU622 and ARU623, which were resistant to amikacin. The *mcr-1* gene, associated with polymyxin resistance, was not identified in any of the strains.

**TABLE 2:** Resistance genes and amino acid changes as determined by whole-genome sequencing analyzed with the ResFinder database and CLC Main Workbench 8.1^*a*^

Gene product	Strain			
	ARU617	ARU620	ARU622	ARU623
β-Lactamases	*bla*_OXA-50_ (T16A, Q25R) *bla*_PAO_ (-) *bla*_TEM-1C_ (-)	*bla*_OXA-50_ (T16A, Q25R) *bla*_PAO_ (P7S, G27D, T105A, V205L, V356I, G391A)	*bla*_OXA-50_ (R49C, D109E, R167H) *bla*_PAO_ (-)	*bla*_OXA-10_ (-) *bla*_OXA-50_ (R49C, D109E, R167H) *bla*_PAO_ (R79Q, T105A)
Aminoglycoside-modifying enzymes	*aph(3′)-IIb (*A107V, E243A)	*aph(3′)-IIb* (A21V, P184L, D188N)	*aac(6′)-Ib* (-) *aph(3′)-IIb* (S14A) *aph(6)-Id* (-)	*aac(6′)-Ib* (-) *aadA3* (-) *aph(3′)-IIb* (S14A) *aph(6)-Id* (-)
Glutathione transferase	*fosA* (-)	*fosA* (-)	*fosA* (-)	*fosA* (-)
Chloramphenicol *O*-acetyltransferase	*catB7* (-)	*catB7* (V97A, G100D, M176I, T195A)	*catB7* (-)	*catB7* (-)

a P. aeruginosa PAO1 was used as a reference (NCBI reference sequence NC_002516). -, no amino acid change detected.

Disruption in the gene encoding the OprD porin was found in the three meropenem-resistant strains (Table 3). Active MexEF-OprN efflux was suspected in all strains, as intact *mexT* sequences were identified (16). Inactivation of the PA3271 gene, an MexEF-OprN activator, was found in ARU620 (17). Previously known mutations in other regulators, e.g., NalC (G71E and S209R) and MexR (V126E), were found in all strains, suggesting MexAB-OprM hyperactivation (18). The MexXY-OprM efflux pump was potentially active in ARU617 and ARU620 due to dysfunctional *mexZ* genes (18). Sequence alterations in the *armZ* gene, encoding a MexZ repressor associated with aminoglycoside resistance, were detected in all strains (19, 20). Finally, an I260V mutation in the MexXY-OprM activator AmgS was found in ARU617 and ARU620 (17). No sequence alteration was identified in the MexCD-OprJ efflux pump repressor NfxB.

**TABLE 3:** Amino acid changes in regulators and subunits of porin OprD and efflux pumps associated with resistance^*a*^

Strain	Porin/efflux pumps, genes, and functions																									
	OprD	MexEF-OprN and MexXY-OprM		MexEF-OprN								MexAB-OprM						MexCD-OprJ				MexXY-OprM				
	*oprD*, S	*parR*, A	*parS*, A	*mexS*, A	*mexT*, A	*cmrA*, R	*mvaT*, R	*PA3271*, A	*mexE*, S	*mexF*, S	*oprN*, S	*mexR*, R	*nalC*, R	*nalD*, R	*mexA*, S	*mexB*, S	*oprM*, S	*nfxB*, R	*mexC*, S	*mexD*, S	*oprJ*, S	*mexZ*, R	*armZ*, R	*amgS*, A	*mexX*, S	*mexY*, S
ARU617	D43N		H398R^*d*^	D249N^*d*^	L26V^*d*^	S26L		A212V^*d*^					G71E^*c*^						S330A^*d*^	E257Q	M69V	D182T^*b*^	L88P^*d*^	I260V	H119Y	T543A^*d*^
	S57E					R29K														S845A	Q267R		D161G		K329Q^*d*^	
	S59R					R33S																	H182Q		W358R^*d*^	
	E202Q					I36V																	V243A^*d*^			
	I210A					E42D																				
	E230K																									
ARU620	S57E	L153R	H398R^*d*^	V73A	L26V^*d*^	T5A		A212V^*d*^			A4T	V126E^*c*^	G71E^*c*^				D448N		S330A^*d*^	S845A		F192A^*b*^	C40R	I260V	K329Q^*d*^	I536V
	S59R	S170N		D249N^*d*^				S602G^*b*^			S13P		A145V										L88P^*d*^		L331V	T543A^*d*^
	V127L												S209R^*c*^										S112N		W358R^*d*^	G589A
	E185Q																						D119E			Q840E
	P185G																						D207N			N1036T
	V189T																						I237V			Q1039R
	E202Q																						V243A^*d*^			
	I210A																									
	E230K																									
	Y236Stop																									
ARU622	T181R^*b*^		H398R^*d*^	T236P	L26V^*d*^	I36V		A212V^*d*^	S8F		S13P	F93I	S209R^*c*^			S1041E			S330A^*d*^	R540G	M69V		L88P^*d*^		K329Q^*d*^	Q282R
				D249N^*d*^		E42D						V126E^*c*^				V1042A				S845A			D161G		W358R^*d*^	T543A^*d*^
																							H182Q			
																							V243A^*d*^			
ARU623	T181R^*b*^		H398R^*d*^	T236P	L26V^*d*^	I36V		A212V^*d*^	S8F		S13P	F93I	S209R^*c*^			S1041E			S330A^*d*^	R540G	M69V		L88P^*d*^		K329Q^*d*^	Q282R
				D249N^*d*^		E42D						V126E^*c*^				V1042A				D657A^*b*^			D161G		W358R^*d*^	T543A^*d*^
																							H182Q			
																							V243A^*d*^			

a P. aeruginosa PAO1 (NCBI reference sequence NC_002516) was used as a reference except for *mexT*, for which P. aeruginosa PAO1-Geneva (GenBank accession number AJ007825.1) was used instead. Abbreviations: S, subunit; A, activator; R, repressor.

b Dysfunctional gene product resulting from frameshift.

c Previously known mutation associated with hyperactive efflux.

d Genetic differences shared between all four strains more likely reflecting variations in the reference sequence.

### Time-lapse microscopy experiments.

In the screening, enhanced activity with the combination compared with the most active single antibiotic, based on the assessment of bacterial density at 24 h, was found in 39 of the 52 (75%) experimental setups (Table 4; see also Table S1 in the supplemental material). All antibiotics demonstrated positive interactions in combination with polymyxin B against at least one strain. Polymyxin B combinations with aztreonam, cefepime, fosfomycin, linezolid, minocycline, thiamphenicol, and trimethoprim were the most promising, showing positive interactions against all four strains. Combinations with meropenem and temocillin were superior to the single drugs at the same concentrations against three of the four strains.

**TABLE 4:** Summary of results^*a*^

Strain	Effect of antibiotic used in combination with polymyxin B												
	AMK	ATM	FEP	CHL	CIP	FOF	LIN	MEM	MIN	RIF	TMC	THI	TMP
ARU617		S*	S*			**S**			S*				
ARU620	S	S*	S*	S*		A		S*	S	S*		**A**	
ARU622		A	**S**			S*		A*				**S**	
ARU623		**A**	**A**		S	A		A*	**S**	S*		A	**A**

a Polymxycin B combinations showing positive interactions in the time-lapse microscopy experiments are highlighted in gray. Additive and synergistic effects at one or several time points in the time-kill experiments are indicated by A and S, respectively. Additive and synergistic interactions at 24 h are in bold. Bactericidal effects at 24 h are marked with an asterisk.

### Time-kill experiments.

**FIG 1:**
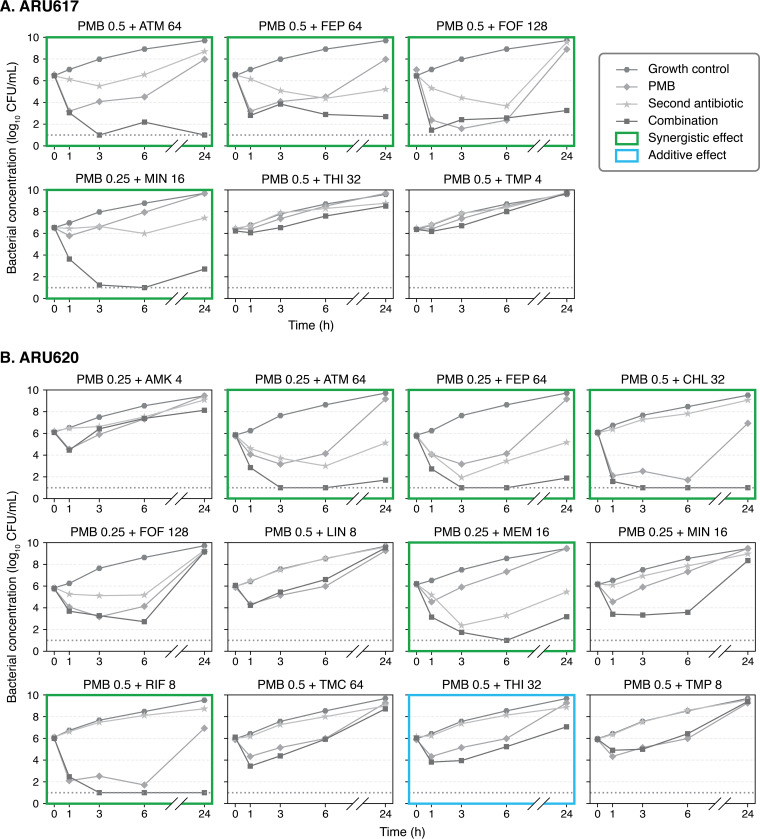

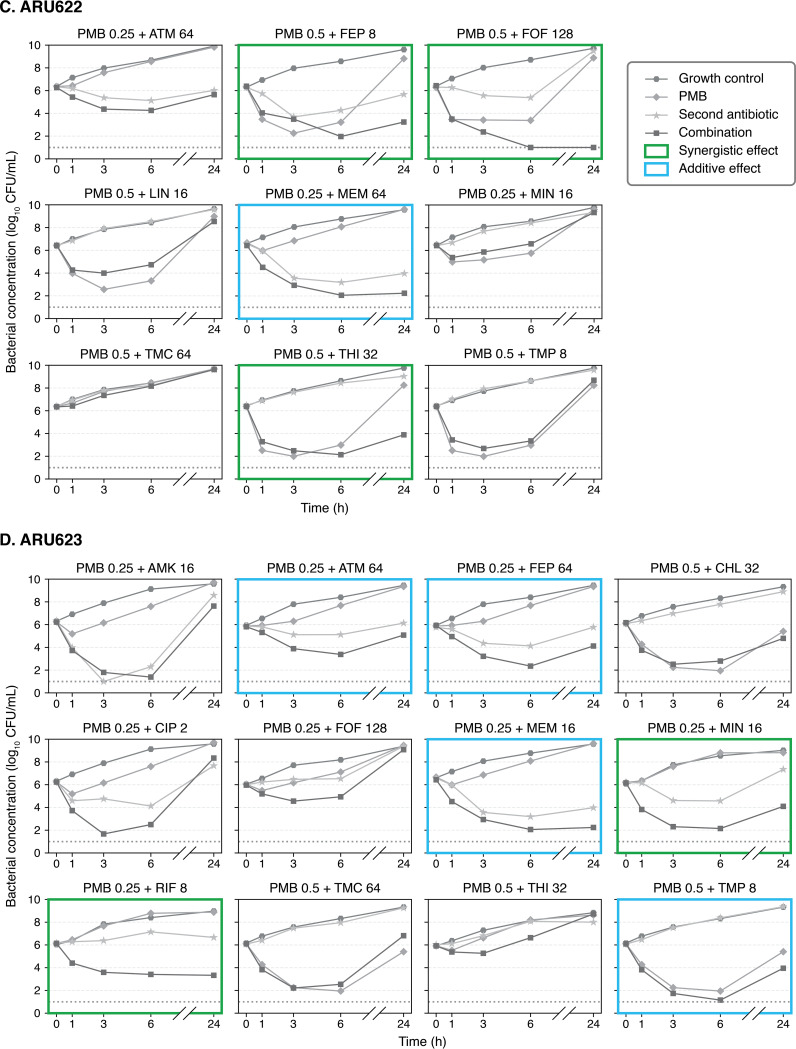
Results of time-kill experiments. Mean bacterial concentrations during 24-h exposure to single antibiotics and polymyxin B combinations at various drug concentrations (in mg/liter) are shown. The lower limit of detection (dotted line) was 10 CFU/ml. Abbreviations: AMK, amikacin; ATM, aztreonam; FEP, cefepime; CHL, chloramphenicol; CIP, ciprofloxacin; FOF, fosfomycin; LIN, linezolid; MEM, meropenem; MIN, minocycline; PMB, polymyxin B; RIF, rifampin; TMC, temocillin; THI, thiamphenicol; TMP, trimethoprim.

Additive or synergistic activity at one or several time points was detected for 27 of the 39 (69%) combinations evaluated in time-kill experiments (Table 4 and Fig. 1; see also Table S2). Such interactions were found with combinations including aztreonam, cefepime, and fosfomycin against all four strains and with meropenem, minocycline, and thiamphenicol against three strains. However, because of continued bacterial killing with one of the single antibiotics or regrowth with the combination later during experiments, positive interactions were less frequently noted by the end of experiments. At 24 h, 20 combinations showed persistent additive (*n* = 6) or synergistic (*n* = 14) effects. Bactericidal activity was achieved with 2 of the additive combinations and 10 of the synergistic combinations.

## DISCUSSION

This study has shown positive interactions of multiple antibiotic combinations against MDR P. aeruginosa in time-lapse microscopy and time-kill experiments. Overall, polymyxin B combinations including aztreonam, cefepime, or meropenem were the most active, indicating an additive or synergistic activity at 24 h in the time-kill experiments against at least three of the four strains used in the study. Fosfomycin, minocycline, and rifampin showed 24-h synergy against two of the four strains.

Resistance in P. aeruginosa is usually multifactorial, entailing decreased permeability of the bacterial outer membrane, increased efflux, and enzymatic activity (2–5). Several resistance genes and mutations associated with resistance were found in the strains used in this study. For example, a carbapenem-resistant phenotype was associated with decreased permeability due to a nonfunctioning *oprD* gene, potential efflux by MexAB-OprM hyperactivation, and genes encoding β-lactamases, e.g., *bla*_PAO_ (Tables 1 to 3) (2, 3). Positive interactions were found with polymyxin B and meropenem against all three strains with a carbapenem-resistant phenotype (Table 4). This finding is in line with previous *in vitro* studies, demonstrating synergy with this combination against MDR P. aeruginosa (11, 21).

The membrane-disrupting feature of the polymyxins, which facilitates the entry of a second antibiotic, thereby counteracting decreased permeability and increased efflux, is probably the most important mechanism of synergy for the tested combinations. The identification of intact *mexT* sequences suggests active MexEF-OprN efflux (22). Further, hyperactivation of MexAB-OprM, resulting in efflux of β-lactam and other antibiotics, was suspected in all strains due to previously known mutations in important regulator genes (3, 16). MexXY-OprM specific efflux could potentially be decreased in ARU622 and ARU623, as the repressor *mexZ* gene was intact, but due to shared substrate specificities, antibiotic-specific conclusions on the activity of efflux pumps are difficult to draw (3). Based on its mechanism of action, polymyxin B is less likely to overcome enzymatic resistance to the second antibiotic used in the combination. This might explain the limited activity of amikacin, as all strains carried genes encoding aminoglycoside-modifying enzymes (Table 2), which play a fundamental role in aminoglycoside resistance (23, 24).

Still, increased intracellular concentrations of the second drug might be sufficient to restore some antibacterial activity provided the enzymatic activity is not too high. Additive and synergistic interactions were frequently found with polymyxin B in combination with aztreonam or cefepime (Table 4), despite the presence of β-lactamase genes. In this case, increased permeability of the β-lactam antibiotics was likely sufficient to counteract the hydrolyzing activity of OXA-50 and OXA-10 (2, 4) as well as efflux through the MexAB-OprM efflux pump (3). To our knowledge, synergy has not been reported with polymyxin B in combination with aztreonam or cefepime against P. aeruginosa. However, the combination of colistin and aztreonam has been reported to be superior to monotherapy *in vitro* and *in vivo* against MDR P. aeruginosa (25). Polymyxin B and cefepime previously showed indifferent effects against MDR P. aeruginosa in a checkerboard screening, although a 38% reduction in polymyxin B MICs was noted (26).

Increased permeation of a second antibiotic, achieved by concurrent administration of polymyxin B, also enables increased activity of substances not normally active against P. aeruginosa, e.g., minocycline and rifampin. The drug target of minocycline is highly conserved, and resistance is commonly caused by decreased permeation and increased efflux (27). The addition of polymyxin has previously been reported to increase the intracellular concentrations of minocycline, resulting in synergy against Acinetobacter baumannii (9). In this study, we found synergy with polymyxin B in combination with minocycline against three of four strains in the time-kill experiments (Table 4). Yet, regrowth occurred in one strain and no significant interaction was observed against one strain, which could be the result of emerging resistant subpopulations (28). The combination of polymyxin B and rifampin was synergistic and bactericidal against two strains despite negligible activity during exposure to rifampin alone. The combination of colistin and rifampin has previously been reported to be synergistic against MDR Gram-negative bacteria, e.g., carbapenemase-producing K. pneumoniae (29).

Polymyxin B and fosfomycin demonstrated positive interactions against all four strains and 24-h synergy in time-kill experiments against two strains (Table 4) despite high fosfomycin MICs (≥64 mg/liter) and the presence of *fosA*, which is highly conserved and causes intrinsic resistance in P. aeruginosa (5). In this case, we hypothesize that the positive combination effects could result from simultaneous actions on targets causing disruption of the bacterial membrane, with polymyxin B targeting the lipid A moiety of the membrane component LPS and fosfomycin acting on the UDP-*N*-acetylglucosamine enolpyruvyl transferase (MurA) (30). The combination of polymyxin B and fosfomycin has been reported to be superior to monotherapy against P. aeruginosa in previous *in vitro* studies, although emergence of resistance has been observed (31).

We have previously reported high consistency in results between time-lapse microscopy and time-kill experiments with single-drug antibiotic exposure against E. coli and P. aeruginosa, as well as colistin combinations against E. coli and K. pneumoniae (32, 33, 35). The present study suggests lower agreement between the two methods for P. aeruginosa than for *Enterobacterales*. Variability in bacterial growth patterns was detected also between replicates with both methods during exposure to polymyxin B alone. This phenomenon, likely a result of biological variation as well as heteroresistance, is commonly observed with P. aeruginosa and adds to the inconsistency in results (28, 34). Further to this, there are important differences in growth conditions, working volumes, and limits of detection between methods (33). The time-kill method generates more precise data on bacterial concentrations and reductions in CFU per milliliter with the combination than with the most active single drug. Still, time-lapse microscopy is more efficient than time-kill experiments, and some disagreement in results could be considered acceptable when used for screening purposes to identify combinations of interest for further evaluation. The oCelloScope has some advantages in comparison with checkerboards, e.g., in that it can be used to monitor morphological changes and bacterial growth dynamics during experiments and has a lower limit of detection (33).

This study has several strengths and limitations. A large number of combinations including antibiotics of several classes were evaluated, some of which, to the best of our knowledge, have not previously been tested against MDR P. aeruginosa. We tested multiple antibiotic concentrations in the range of clinically achievable blood concentrations. Still, using additional drug concentrations could have resulted in the detection of more positive interactions, and some of the promising regimens may require concentrations that are potentially toxic *in vivo*. The low number of strains is an important limitation of this study, and more research is needed to determine the general susceptibility of MDR P. aeruginosa to the promising combinations. Regrowth was frequently observed, which could be due to heteroresistance, selection of resistant subpopulations, and antibiotic-induced tolerance. Further evaluation is required to assess the mechanisms of regrowth and the ability of the combinations to suppress emergence of resistance. Genetic characterization of the tested strains was performed by analyzing whole-genome sequencing data for known resistance genes and mechanisms, which provided some insights and hypotheses on potential mechanisms of interaction. Such data can also be valuable in the future understanding of the variability in results between studies with regard to the activity of antibiotic combinations and genotype-phenotype associations.

In conclusion, we have demonstrated positive interactions with multiple polymyxin B combinations against MDR P. aeruginosa, most frequently with aztreonam, cefepime, and meropenem. Additive and synergistic activities were sometimes observed also in the presence of resistance determinants to the single drugs and with antibiotics that are normally inactive against P. aeruginosa. Further study is required to gain a better understanding of the mechanisms of synergy, pharmacokinetic-pharmacodynamic determinants of efficacy, and potential clinical benefits of the promising antibiotic combinations.

## MATERIALS AND METHODS

### Strains, media, and antibiotic susceptibility testing.

Four clinical isolates of P. aeruginosa, provided by the Public Health Agency of Sweden, were included in the study. Cation-adjusted Mueller-Hinton broth and agar plates (BD Diagnostics, Franklin Lakes, NJ) were used in all experiments. Glucose-6-phosphate was added to 25 mg/liter in experiments with fosfomycin (13). The MICs were determined in duplicates using the agar dilution method for fosfomycin and the broth microdilution method for polymyxin B and thiamphenicol according to EUCAST guidelines (13) and the gradient test method (Etest; bioMérieux, Marcy-l’Étoile, France) for the other antibiotics. If discrepant results were found, a third replicate was performed and the median MIC value was presented.

### Antibiotics.

Antibiotics were selected to represent multiple classes and to include antibiotics that are often prescribed for P. aeruginosa infections as well as drugs that are normally inactive against Gram-negative bacteria but could potentially be useful in combination. Purchases were made from Sigma-Aldrich (St. Louis, MO), except for temocillin, which was kindly provided by Eumedica S.A. (Manage, Belgium). Stock solutions of 10,000 mg/liter of active substance were prepared according to the manufacturers’ instructions. Further dilutions were prepared in sterile water and stored at –20°C for up to 30 days. For polymyxin B, stock solutions of 10 mg/liter, 100 mg/liter, and 1,000 mg/liter were prepared directly from the 10,000-mg/liter stock using 50-ml polypropylene Falcon tubes (Thermo Fisher Scientific Inc., Waltham, MA) to reduce plastic binding of the compound.

### Genetic characterization.

DNA was extracted by the MagNA Pure 96 system (F. Hoffmann-La Roche, Basel, Switzerland), and whole-genome sequencing was performed using the HiSeq platform (Illumina Inc., San Diego, CA). FastQC (Babraham Institute, Cambridge, UK) was employed for quality check before *de novo* assembly was performed using CLC Genomics Workbench version 11 (Qiagen, Hilden, Germany). Contigs were subjected to analysis by the ResFinder database (14) to identify resistance genes, followed by verification in GenBank (National Center for Biotechnology Information, Bethesda, MD). CLC Main Workbench 8.1 (Qiagen) was employed for a comparison with P. aeruginosa PAO1 (NCBI reference sequence NC_002516) sequences of porins, efflux pumps, and regulators frequently associated with resistance in P. aeruginosa: OprD, MexAB-OprM, MexCD-OprJ, MexEF-OprN, and MexXY-OprM (17). P. aeruginosa PAO1-geneva (GenBank accession number AJ007825.1) was used as a reference strain for *mexT* because P. aeruginosa PAO1 has been reported to have nonfunctional *mexT* (22).

### Time-lapse microscopy experiments.

Screening was performed using the oCelloScope instrument as previously described (33). Bacteria were added to achieve starting inocula of 10^6^ CFU/ml and a total volume of 200 μl in a 96-well microtiter plate. The bacteria were exposed to single antibiotics and combinations of polymyxin B and a second drug at the following concentrations: polymyxin B, 0.25, 0.5, 1, and 2 mg/liter; amikacin, 4, 16, and 128 mg/liter; aztreonam, 2, 8, and 64 mg/liter; cefepime, 2, 8, and 64 mg/liter; chloramphenicol, 1, 8, and 32 mg/liter; ciprofloxacin, 0.25, 2, and 8 mg/liter; fosfomycin, 8, 32, and 128 mg/liter; linezolid, 2, 8, and 16 mg/liter; meropenem, 2, 16, and 64 mg/liter; minocycline, 0.5, 4, and 16 mg/liter; rifampin, 1, 8, and 32 mg/liter; temocillin, 4, 16, and 64 mg/liter; thiamphenicol, 2, 8, and 32 mg/liter; and trimethoprim, 1, 4, and 8 mg/liter. The oCelloScope was placed in a 37°C incubator for 24 h, and five images were acquired for each well every 15 min. Focus was set using the bottom search function. All experiments were performed in duplicate.

The UniExplorer software (version 6.0) algorithms were used to calculate background corrected absorption (BCA) and segmentation and extraction of surface area (SESA). BCA at >8.0 at 24 h and a maximum SESA value (SESA_max_) at >5.8 were applied as cutoff values, indicating a bacterial density of >10^6^ CFU/ml in the wells after 24 h (33). If the 24-h BCA and/or SESA_max_ values were below the cutoffs with the combination but not with either of the single antibiotics at the same concentration, the combination was considered to show a positive interaction and was subjected to subsequent evaluation in time-kill experiments.

### Time-kill experiments.

A polymyxin B concentration of 0.5× MIC was used in most experiments to avoid extensive killing by polymyxin B alone. However, if a concentration >0.5× MIC was required to achieve a positive interaction in the screening, that specific concentration was used in the time-kill experiments. The second antibiotic was added to achieve the highest concentration showing enhanced activity in combination in the time-lapse microscopy experiments. A starting inoculum of approximately 5 × 10^6^ CFU/ml was prepared as previously described (33) to a total volume of 2.5 ml. Aliquots were obtained at 0, 1, 3, 6, and 24 h, serially diluted, and spread on plates. Colonies were counted after 18 to 24 h of incubation at 37°C. Bacterial counts below the lower limit of detection (10 CFU/ml) were counted as 1 log_10_ CFU/ml. All experiments were performed in duplicate, and mean values were used in the analysis. Synergy was defined as a ≥2-log_10_ decrease in CFU per milliliter with the combination, compared with the most active single antibiotic, and an additive effect was defined as a 1- to 2-log_10_ reduction in CFU per milliliter with the combination. A bactericidal effect was defined as a ≥3-log_10_ decrease in CFU per milliliter after 24 h compared with the starting inoculum.

## Supplementary Material

Supplemental file 1
