# Supplementary material for: Efficacy of Antibiotic Combinations against Multidrug-Resistant Pseudomonas aeruginosa in Automated Time-Lapse Microscopy and Static Time-Kill Experiments
Source: Antimicrob Agents Chemother. 2020 May 21;64(6):e02111-19. doi: 10.1128/AAC.02111-19 (PMC7269485; doi:10.1128/AAC.02111-19)
Supplement: Supplemental file 1 [file AAC.02111-19-s0001.pdf]

Supplementary data.

Table S1. Results of time-lapse microscopy experiments with polymyxin B and 13 other antibiotics, alone and in combination, at various concentrations (mg/L). Background Corrected Absorption (BCA) at 24 h and the maximum Segmentation and Extraction of Surface Area ($SESA_{max}$) of duplicate experiments are presented. BCA >8.0 at 24 h and $SESA_{max}$ >5.8 were used as cut-offs indicating bacterial density >10^6 CFU/mL (highlighted in grey). Combinations that prevented bacterial growth at 24 h while none of the single antibiotics did were subsequently evaluated in the time-kill experiments at the concentrations indicated in bold.

Supplementary data.

**Amikacin**		AMK 0	AMK 4	AMK 16	AMK 128	AMK 0	AMK 4	AMK 16	AMK 128
**ARU617**	PMB 0	6.17 9.02	6.28 8.94	4.46 8.13	5.11 7.45	6.19 9.02	6.27 8.77	4.98 6.66	4.09 7.21
	PMB 0.25	6.22 9.02	6.27 8.96	4.90 8.35	4.82 7.87	6.19 9.04	6.29 8.59	4.31 4.67	3.91 5.40
	PMB 0.5	4.03 5.06	4.68 7.04	4.40 7.80	4.13 7.42	4.74 6.78	3.61 5.15	4.50 6.09	4.51 5.95
	PMB 1	4.34 5.55	4.35 6.30	4.23 6.97	4.59 6.37	3.93 6.39	4.29 5.88	4.26 5.90	4.59 6.49
	PMB 2	4.17 5.90	3.98 6.30	4.21 6.13	4.01 6.89	4.41 7.20	4.55 5.53	4.06 5.60	4.56 6.31
**ARU620**	PMB 0	5.91 9.07	6.18 8.95	5.28 6.61	5.10 6.78	5.99 9.02	6.20 8.95	5.65 6.71	5.44 6.43
	PMB 0.25	5.95 9.07	**5.54 7.13**	5.19 6.02	5.09 6.04	6.05 9.02	**5.49 6.69**	5.48 6.08	5.33 5.95
	PMB 0.5	5.00 6.74	5.07 5.88	5.16 6.20	5.17 6.13	6.17 9.07	5.47 7.38	5.37 6.13	5.43 6.25
	PMB 1	4.80 5.99	4.91 7.29	5.03 6.67	5.04 6.24	5.20 6.82	5.23 6.53	5.27 6.80	5.32 7.02
	PMB 2	4.78 6.37	4.88 6.26	4.86 6.27	4.98 6.24	5.19 7.80	5.20 7.27	5.25 7.60	5.28 7.33
**ARU622**	PMB 0	6.08 9.01	6.18 9.00	5.12 5.64	5.16 6.02	6.09 9.07	4.99 6.67	4.92 6.88	4.80 6.12
	PMB 0.25	6.09 9.03	6.13 8.98	5.07 5.80	5.03 5.79	6.11 9.07	4.78 5.89	3.89 5.18	4.71 5.50
	PMB 0.5	5.07 6.18	4.99 5.35	5.12 5.53	4.74 5.52	4.79 6.64	4.80 6.37	4.60 6.02	4.62 6.18
	PMB 1	5.37 7.16	5.00 5.51	4.97 5.85	4.85 6.05	4.57 6.05	4.53 5.54	4.32 5.96	4.54 6.26
	PMB 2	4.35 5.56	4.83 5.85	5.01 6.00	4.95 5.81	4.23 7.02	4.48 5.95	4.61 6.12	4.67 6.22
**ARU623**	PMB 0	6.02 9.05	6.23 8.99	6.13 8.83	4.70 6.39	6.06 9.03	6.12 8.98	4.82 6.48	4.77 7.07
	PMB 0.25	6.16 8.95	6.23 8.98	**4.72 5.56**	4.78 5.64	6.20 8.98	6.12 8.97	4.57 5.22	4.29 5.59
	PMB 0.5	5.96 8.95	4.85 6.50	4.31 6.20	4.76 6.02	6.10 8.81	5.11 6.95	3.93 5.85	4.87 6.62
	PMB 1	4.33 6.04	4.44 6.29	4.31 7.00	4.83 6.10	6.10 8.74	4.66 7.08	4.79 7.27	4.07 7.62
	PMB 2	4.00 6.14	4.63 6.97	4.36 6.41	4.77 6.91	4.02 6.78	4.44 6.12	4.66 7.55	4.83 7.28
**Aztreonam**		ATM 0	ATM 2	ATM 8	ATM 64	ATM 0	ATM 2	ATM 8	ATM 64
**ARU617**	PMB 0	6.20 9.06	6.23 9.01	6.26 8.96	6.28 8.62	6.19 9.05	6.20 9.00	6.29 8.93	6.27 8.68
	PMB 0.25	6.19 9.02	6.12 9.02	6.29 8.96	6.26 8.59	6.27 9.03	6.26 9.00	6.31 8.82	5.47 7.24
	PMB 0.5	4.81 6.11	6.26 8.92	4.68 6.27	4.68 6.18	6.02 8.54	6.36 8.89	5.64 7.71	**4.71 6.43**
	PMB 1	4.47 6.44	4.64 6.29	4.05 5.62	4.24 6.18	5.04 7.91	4.67 6.48	5.91 8.21	4.63 6.55
	PMB 2	4.28 6.09	4.33 5.61	4.32 6.38	4.26 5.87	4.66 6.90	4.58 6.97	4.61 6.92	4.64 6.78
**ARU620**	PMB 0	6.02 8.99	6.25 8.99	6.14 9.00	5.92 8.00	6.07 9.07	6.24 9.01	6.06 7.81	6.03 8.05
	PMB 0.25	6.20 8.98	6.20 8.98	6.01 9.01	5.38 5.69	6.08 9.02	6.24 8.98	5.32 7.01	**5.23 6.26**
	PMB 0.5	5.90 8.77	4.83 5.85	4.83 6.38	4.92 6.54	5.10 8.65	4.63 6.57	5.00 6.62	5.15 7.00
	PMB 1	4.50 7.76	4.69 6.96	4.65 7.74	4.82 8.02	4.85 7.41	5.72 8.63	5.01 8.19	4.91 8.27
	PMB 2	4.33 8.09	4.83 7.71	4.30 8.37	4.52 8.15	4.70 8.44	4.84 8.60	4.85 8.70	4.98 8.61
**ARU622**	PMB 0	6.05 9.07	6.10 9.03	6.23 9.05	6.25 8.65	5.81 9.03	6.15 9.00	6.22 8.99	6.28 8.33
	PMB 0.25	6.08 9.07	6.10 9.01	6.13 9.01	**5.44 6.84**	6.10 9.04	6.15 8.99	6.15 9.01	**5.87 6.53**
	PMB 0.5	4.93 7.74	6.06 8.67	5.06 6.66	5.10 6.59	5.90 9.00	6.12 9.00	5.21 6.12	5.24 6.34
	PMB 1	4.92 8.32	4.52 6.10	5.00 6.79	5.12 6.79	5.80 8.76	5.79 7.74	5.10 7.20	5.13 7.71
	PMB 2	4.64 6.53	4.89 6.77	4.86 7.83	4.96 7.10	4.72 8.42	4.94 8.17	4.96 7.76	4.92 8.18
**ARU623**	PMB 0	6.02 8.99	6.25 8.99	6.14 9.00	5.92 8.00	6.18 8.99	6.15 8.98	6.20 8.93	5.89 8.13
	PMB 0.25	6.20 8.98	6.20 8.98	6.01 9.01	5.38 5.69	5.99 9.02	6.17 8.96	6.07 8.94	**5.43 5.94**
	PMB 0.5	5.90 8.77	4.83 5.85	4.83 6.38	4.92 6.54	4.97 7.52	5.78 8.38	4.88 6.06	4.93 6.41
	PMB 1	4.50 7.76	4.69 6.96	4.65 7.74	4.82 8.02	4.58 6.72	4.61 7.10	4.76 7.39	4.79 7.81
	PMB 2	4.33 8.09	4.83 7.71	4.30 8.37	4.52 8.15	4.48 8.31	4.80 8.60	4.23 8.57	4.59 8.21

Supplementary data.

Cefepime		FEP 0		FEP 2		FEP 8		FEP 64		FEP 0		FEP 2		FEP 8		FEP 64	
**ARU617**	PMB 0	6.20	9.06	6.25	9.01	6.32	8.77	6.03	7.78	6.19	9.05	6.23	9.01	6.30	8.75	6.20	8.52
	PMB 0.25	6.19	9.02	6.25	9.01	6.31	8.81	5.96	7.44	6.27	9.03	6.25	9.01	6.30	8.72	5.96	7.60
	PMB 0.5	4.81	6.11	4.24	6.08	4.38	6.18	4.54	6.54	6.02	8.54	4.85	6.71	4.89	6.70	**4.59**	**6.90**
	PMB 1	4.47	6.44	4.58	6.65	4.30	6.55	4.44	6.09	5.04	7.91	4.75	6.76	4.78	7.12	4.72	6.99
	PMB 2	4.28	6.09	4.33	6.17	4.47	6.18	4.52	6.23	4.66	6.90	4.67	6.78	4.86	6.94	4.68	6.76
**ARU620**	PMB 0	6.02	8.99	6.17	9.00	6.15	9.06	5.96	8.20	6.07	9.07	6.28	8.98	6.17	8.29	6.06	7.83
	PMB 0.25	6.20	8.98	6.03	8.99	6.09	8.75	**5.49**	**7.17**	6.08	9.02	6.29	8.97	5.24	7.37	5.20	7.19
	PMB 0.5	5.90	8.77	4.62	7.42	4.99	7.77	4.59	7.49	5.10	8.65	6.10	8.65	5.15	8.04	5.08	7.94
	PMB 1	4.50	7.76	4.45	7.91	4.45	7.93	4.79	7.81	4.85	7.41	5.03	8.47	4.95	8.18	5.05	8.17
	PMB 2	4.33	8.09	4.59	8.10	4.23	7.93	4.64	7.61	4.70	8.44	5.01	8.51	4.99	8.11	4.91	8.00
**ARU622**	PMB 0	6.05	9.07	6.26	9.05	6.25	8.73	5.86	7.56	5.81	9.03	6.22	8.97	6.28	8.90	6.17	7.62
	PMB 0.25	6.08	9.07	6.17	9.01	6.21	8.04	6.06	7.77	6.10	9.04	6.18	8.99	6.23	8.91	5.73	6.83
	PMB 0.5	4.93	7.74	4.86	6.87	5.21	7.22	5.14	7.22	5.90	9.00	5.22	6.90	**5.22**	**7.26**	5.23	7.03
	PMB 1	4.92	8.32	5.11	7.06	5.09	6.85	5.05	7.13	5.80	8.76	5.13	7.98	5.13	7.84	5.05	7.49
	PMB 2	4.64	6.53	4.78	8.19	4.90	8.29	4.97	8.34	4.72	8.42	4.54	7.30	4.96	7.66	4.92	7.39
**ARU623**	PMB 0	6.02	8.99	6.17	9.00	6.15	9.06	5.96	8.20	6.18	8.99	6.12	8.98	6.25	8.90	5.91	8.27
	PMB 0.25	6.20	8.98	6.03	8.99	6.09	8.75	**5.49**	**7.17**	5.99	9.02	6.16	8.96	5.59	6.87	**5.17**	**6.74**
	PMB 0.5	5.90	8.77	4.62	7.42	4.99	7.77	4.59	7.49	4.97	7.52	4.77	7.00	4.59	6.78	4.91	7.41
	PMB 1	4.50	7.76	4.45	7.91	4.45	7.93	4.79	7.81	4.58	6.72	4.81	8.19	4.72	7.88	4.73	7.74
	PMB 2	4.33	8.09	4.59	8.10	4.23	7.93	4.64	7.61	4.48	8.31	4.11	8.06	4.66	7.24	4.49	7.64
**Chloramphenicol**		CHL 0		CHL 1		CHL 8		CHL 32		CHL 0		CHL 1		CHL 8		CHL 32	
**ARU617**	PMB 0	6.17	9.02	6.23	9.03	6.24	9.01	6.20	9.00	6.19	9.02	6.26	9.01	6.27	9.01	6.28	8.98
	PMB 0.25	6.22	9.02	6.20	9.03	6.21	9.02	6.24	9.00	6.19	9.04	6.24	9.00	6.24	9.00	6.27	8.96
	PMB 0.5	4.03	5.06	4.33	7.50	4.46	7.25	4.33	6.94	4.74	6.78	4.50	6.19	4.53	6.13	4.74	6.31
	PMB 1	4.34	5.55	4.27	6.56	3.89	6.95	4.11	6.48	3.93	6.39	4.37	6.10	4.61	6.31	4.57	6.41
	PMB 2	4.17	5.90	4.22	6.85	4.30	6.61	4.18	5.81	4.41	7.20	4.23	6.20	4.47	6.48	4.38	6.39
**ARU620**	PMB 0	5.91	9.07	6.07	9.07	6.07	9.07	6.09	9.05	5.99	9.02	6.16	9.00	6.16	8.99	6.14	9.06
	PMB 0.25	5.95	9.07	6.10	8.99	6.13	8.98	6.26	8.89	6.05	9.02	6.13	9.00	6.18	8.98	6.16	9.04
	PMB 0.5	5.00	6.74	5.17	6.45	5.10	6.29	5.18	6.46	6.17	9.07	5.49	7.19	5.53	6.98	**5.55**	**6.71**
	PMB 1	4.80	5.99	5.17	6.45	5.04	6.76	4.77	6.13	5.20	6.82	5.40	7.21	5.36	6.80	5.26	6.89
	PMB 2	4.78	6.37	5.02	6.69	4.94	6.51	4.84	6.41	5.19	7.80	5.26	6.31	5.30	7.04	5.42	6.64
**ARU622**	PMB 0	6.08	9.01	6.12	9.01	6.14	9.01	6.19	8.99	6.09	9.07	6.12	9.02	6.11	9.01	6.12	9.06
	PMB 0.25	6.09	9.03	6.12	9.01	6.16	9.00	6.15	8.98	6.11	9.07	6.03	9.02	6.11	9.02	6.19	8.99
	PMB 0.5	5.07	6.18	5.28	6.08	6.31	8.85	5.23	6.34	4.79	6.64	4.87	6.92	4.81	6.74	4.86	6.36
	PMB 1	5.37	7.16	5.20	5.88	5.04	6.04	5.04	6.46	4.57	6.05	4.43	6.59	4.59	6.55	4.69	6.43
	PMB 2	4.35	5.56	4.83	5.92	4.75	5.76	4.58	6.33	4.23	7.02	4.37	6.38	4.62	6.99	4.61	6.17
**ARU623**	PMB 0	6.02	9.05	6.27	8.93	6.19	8.93	6.13	8.97	6.06	9.03	6.23	8.98	6.26	8.96	6.24	8.96
	PMB 0.25	6.16	8.95	6.26	8.93	6.27	8.92	6.26	8.95	6.20	8.98	6.21	8.98	6.26	8.96	6.18	8.96
	PMB 0.5	5.96	8.95	5.02	6.65	4.93	6.33	**4.81**	**6.42**	6.10	8.81	4.58	6.76	4.99	7.58	**5.06**	**7.27**
	PMB 1	4.33	6.04	4.80	7.12	4.68	6.39	4.63	6.30	6.10	8.74	4.90	7.69	4.86	6.79	4.88	6.54
	PMB 2	4.00	6.14	4.50	7.29	4.73	6.46	5.91	8.22	4.02	6.78	4.67	7.00	4.82	6.70	4.74	6.26

Supplementary data.

Ciprofloxacin		CIP 0	CIP 0.25	CIP 2	CIP 8	CIP 0	CIP 0.25	CIP 2	CIP 8
	PMB 0	6.17 9.02	4.08 7.33	6.27 8.62	4.85 7.74	6.19 9.02	6.21 9.03	6.11 8.05	5.36 6.82
	PMB 0.25	6.22 9.02	6.21 9.02	6.28 8.61	4.99 7.89	6.19 9.04	6.22 9.01	6.29 8.57	4.76 6.01
**ARU617**	PMB 0.5	4.03 5.06	4.49 7.07	4.52 7.22	4.42 7.60	4.74 6.78	6.30 8.59	4.77 6.60	4.63 6.72
	PMB 1	4.34 5.55	4.45 6.70	4.48 6.66	3.99 7.00	3.93 6.39	4.24 5.51	3.96 6.12	4.54 6.67
	PMB 2	4.17 5.90	4.16 6.32	6.20 8.69	4.28 6.55	4.41 7.20	4.76 6.49	4.59 6.33	4.38 6.17
	PMB 0	5.91 9.07	6.09 9.07	6.07 9.06	5.78 8.02	5.99 9.02	6.18 9.00	6.28 8.95	5.91 7.73
	PMB 0.25	5.95 9.07	6.10 9.00	6.26 8.95	5.39 6.66	6.05 9.02	6.11 9.01	6.27 8.95	6.07 8.02
**ARU620**	PMB 0.5	5.00 6.74	4.97 5.36	5.17 6.28	5.20 6.43	6.17 9.07	6.16 9.00	6.17 8.95	6.12 8.41
	PMB 1	4.80 5.99	5.02 6.53	4.93 6.52	5.00 6.41	5.20 6.82	5.30 6.87	5.39 6.94	5.38 7.02
	PMB 2	4.78 6.37	4.90 6.44	4.98 6.24	4.92 6.78	5.19 7.80	5.28 6.71	5.36 6.83	5.28 6.96
	PMB 0	6.08 9.01	6.17 9.01	6.27 8.92	6.16 8.94	6.09 9.07	6.11 9.07	6.15 9.02	5.57 7.01
	PMB 0.25	6.09 9.03	6.17 9.00	6.28 8.89	6.28 8.87	6.11 9.07	6.14 9.01	6.29 8.88	6.04 8.84
**ARU622**	PMB 0.5	5.07 6.18	4.80 6.41	5.13 6.02	5.20 6.18	4.79 6.64	4.76 6.80	4.70 6.50	4.88 6.93
	PMB 1	5.37 7.16	5.06 6.21	5.07 6.02	5.02 6.01	4.57 6.05	4.63 6.27	4.71 6.22	4.26 6.33
	PMB 2	4.35 5.56	4.91 6.03	4.93 6.08	4.99 6.23	4.23 7.02	4.57 6.52	4.68 6.66	4.43 6.86
	PMB 0	6.02 9.05	6.26 8.89	6.07 8.51	5.22 7.46	6.06 9.03	6.23 8.97	6.24 8.92	5.37 7.99
	PMB 0.25	6.16 8.95	6.27 8.90	**5.12 6.87**	5.10 6.64	6.20 8.98	6.26 8.96	6.17 8.92	5.31 7.06
**ARU623**	PMB 0.5	5.96 8.95	4.96 6.59	4.61 6.48	5.01 7.24	6.10 8.81	5.05 7.32	5.05 7.01	5.10 7.23
	PMB 1	4.33 6.04	4.79 6.86	4.82 7.01	4.77 7.08	6.10 8.74	4.85 7.21	4.79 7.31	4.81 7.45
	PMB 2	4.00 6.14	4.60 5.95	4.82 6.72	4.61 6.66	4.02 6.78	4.58 6.53	4.24 6.90	4.81 7.11
**Fosfomycin**		FOF 0	FOF 8	FOF 32	FOF 128	FOF 0	FOF 8	FOF 32	FOF 128
	PMB 0	6.26 9.04	6.28 8.71	6.32 8.70	6.15 8.21	6.26 9.02	6.31 8.67	6.30 8.39	6.27 8.40
	PMB 0.25	6.23 9.04	6.29 8.74	6.30 8.83	5.64 7.67	6.24 9.02	6.31 8.64	6.15 8.23	6.15 8.33
**ARU617**	PMB 0.5	6.28 8.99	6.32 8.85	4.85 6.79	**4.52 6.99**	6.30 8.80	4.91 7.34	4.83 6.88	**5.02 7.57**
	PMB 1	4.65 7.09	4.50 6.91	4.39 7.28	4.60 7.22	4.81 6.86	5.06 7.79	4.92 6.72	5.06 7.64
	PMB 2	3.92 5.95	4.54 6.22	4.68 6.67	4.46 7.02	4.41 7.15	5.04 7.36	4.79 6.60	4.87 7.25
	PMB 0	6.04 9.07	6.17 8.99	5.81 7.92	6.07 9.07	6.06 9.04	6.19 8.99	6.22 8.77	5.90 8.38
	PMB 0.25	6.13 9.03	6.16 9.07	6.02 9.00	**5.11 6.99**	6.10 9.01	6.16 8.98	6.12 8.62	**5.36 7.72**
**ARU620**	PMB 0.5	6.15 8.86	4.83 7.41	4.78 6.86	4.48 8.12	6.17 8.99	5.16 7.47	5.12 7.57	5.03 7.97
	PMB 1	4.62 8.63	4.71 7.59	4.58 7.25	4.46 8.56	4.98 7.54	4.88 8.04	5.02 7.78	4.86 8.69
	PMB 2	4.70 6.92	4.64 7.68	4.43 7.23	4.39 8.51	4.82 6.61	4.88 6.56	5.05 7.98	4.83 8.71
	PMB 0	6.14 8.99	6.25 8.97	6.14 8.97	6.15 8.98	6.11 9.02	6.03 9.01	6.16 9.00	6.14 9.00
	PMB 0.25	6.25 8.98	6.16 8.97	6.16 8.98	6.13 8.94	6.08 9.03	5.96 9.01	6.22 8.95	6.11 8.99
**ARU622**	PMB 0.5	6.12 9.00	5.10 6.91	6.11 8.95	**5.01 6.95**	6.12 9.03	6.05 9.01	4.73 8.32	**4.92 8.35**
	PMB 1	6.10 8.81	5.10 7.16	4.80 7.28	4.86 6.96	4.83 8.48	4.63 8.50	4.72 8.61	4.91 8.59
	PMB 2	6.13 8.55	4.82 6.82	4.83 6.97	4.79 6.69	4.64 8.42	4.71 8.60	4.70 8.45	4.63 8.45
	PMB 0	6.10 9.02	6.23 8.97	6.14 8.97	6.16 8.99	6.17 9.00	6.17 8.99	6.09 9.00	6.00 9.01
	PMB 0.25	6.22 8.98	6.08 9.00	6.05 9.01	5.95 9.02	6.25 9.00	6.13 8.98	6.16 8.99	**4.93 8.65**
**ARU623**	PMB 0.5	5.22 6.76	4.93 6.66	4.88 6.35	4.82 7.18	4.39 7.97	4.46 8.67	4.54 8.75	4.57 8.74
	PMB 1	4.78 8.47	4.83 6.81	4.91 6.91	4.70 7.54	4.73 8.73	4.72 8.69	4.58 8.78	4.62 8.80
	PMB 2	4.74 6.15	4.61 6.80	4.75 7.58	4.70 6.98	4.65 8.30	4.55 8.70	4.53 8.76	4.32 8.82

Supplementary data.

**Linezolid**		LIN 0	LIN 2	LIN 8	LIN 16	LIN 0	LIN 2	LIN 8	LIN 16
**ARU617**	PMB 0	6.23 9.02	6.25 9.01	6.28 9.05	6.26 9.01	6.24 9.06	6.25 9.01	6.26 9.06	6.27 9.06
	PMB 0.25	6.21 9.02	6.27 9.01	6.28 9.01	6.27 9.01	6.24 9.01	6.29 9.00	6.27 9.01	6.27 9.01
	PMB 0.5	6.29 9.00	6.25 9.01	6.28 9.00	6.27 9.01	6.28 8.89	6.29 8.89	6.30 8.87	6.29 8.87
	PMB 1	4.85 6.37	5.22 6.37	4.98 6.79	6.27 8.51	4.95 7.23	5.12 8.04	5.02 7.65	4.96 7.35
	PMB 2	4.43 6.24	5.08 6.69	4.90 6.72	4.92 6.73	4.53 7.07	4.86 7.72	4.83 7.45	4.73 7.33
**ARU620**	PMB 0	5.91 9.07	6.07 9.07	6.18 9.00	6.09 9.07	5.93 9.01	5.99 9.01	5.97 9.00	6.01 9.01
	PMB 0.25	6.06 9.01	6.19 8.99	6.18 8.99	6.03 9.07	5.79 9.00	6.09 9.00	6.06 8.99	5.98 8.99
	PMB 0.5	6.12 9.07	6.15 9.00	6.15 9.00	6.18 8.99	6.08 9.00	5.85 9.07	**4.97 6.75**	5.98 9.00
	PMB 1	5.08 6.64	5.11 7.27	5.05 7.43	4.96 6.72	4.93 6.02	4.41 5.51	5.22 6.19	6.06 8.98
	PMB 2	4.86 6.98	4.94 6.72	5.06 7.73	4.82 6.58	5.05 7.33	5.07 6.51	4.89 6.27	5.03 6.29
**ARU622**	PMB 0	6.09 9.08	6.14 9.07	6.12 9.07	6.12 9.08	6.08 9.03	6.01 9.02	5.98 9.07	6.11 9.02
	PMB 0.25	6.11 9.07	6.16 9.01	6.09 9.07	6.09 9.02	6.08 9.03	5.99 9.01	5.97 9.01	6.10 9.01
	PMB 0.5	6.12 9.07	6.10 9.07	6.02 9.07	**6.12 9.07**	6.03 9.07	6.09 9.01	6.01 9.01	**6.06 8.99**
	PMB 1	6.07 9.07	5.00 7.70	5.84 8.03	5.09 6.92	5.99 8.75	6.08 8.43	5.98 8.30	5.18 7.18
	PMB 2	4.70 7.80	4.97 7.99	4.82 6.90	4.89 7.41	4.90 6.34	5.04 6.45	5.73 7.26	4.69 6.09
**ARU623**	PMB 0	6.13 9.04	6.07 9.05	6.15 9.05	6.10 9.06	5.97 9.06	6.15 9.07	6.15 9.06	6.11 9.06
	PMB 0.25	6.11 9.05	6.01 9.04	6.14 9.03	6.09 9.03	6.03 9.06	6.20 8.97	6.11 8.97	6.12 9.06
	PMB 0.5	6.13 9.05	6.15 9.04	6.13 9.04	6.10 9.03	5.22 7.21	5.96 8.78	4.88 5.84	6.12 8.82
	PMB 1	4.93 7.64	5.95 8.18	4.88 6.73	4.92 6.71	4.52 6.20	4.72 6.51	5.29 7.92	4.86 6.47
	PMB 2	4.85 7.14	4.75 7.44	4.72 7.34	4.70 7.24	4.68 6.17	4.70 6.44	4.57 6.53	4.57 5.97
**Meropenem**		MEM 0	MEM 2	MEM 16	MEM 64	MEM 0	MEM 2	MEM 16	MEM 64
**ARU617**	PMB 0	6.19 9.02	5.93 7.93	5.73 7.58	5.69 8.11	6.21 9.03	5.75 7.72	6.11 8.38	5.45 8.32
	PMB 0.25	6.19 9.04	4.93 8.58	4.88 6.81	4.35 7.06	6.18 9.03	5.28 6.88	4.58 5.98	4.49 5.86
	PMB 0.5	4.74 6.78	4.07 7.87	3.75 6.06	4.62 7.13	6.21 8.85	4.35 5.06	4.01 6.04	4.16 7.08
	PMB 1	3.93 6.39	4.31 6.49	3.93 6.17	4.21 7.51	4.03 8.04	4.10 7.60	4.44 7.21	3.78 8.30
	PMB 2	4.41 7.20	3.87 4.79	4.38 6.07	4.49 6.61	4.24 8.56	4.01 6.32	4.11 8.47	3.94 8.50
**ARU620**	PMB 0	5.91 9.07	6.12 9.07	5.62 7.43	5.45 8.37	5.99 9.02	6.27 8.97	6.07 8.09	5.73 8.47
	PMB 0.25	5.95 9.07	6.08 9.07	5.24 7.20	5.21 7.42	6.05 9.02	6.16 9.00	**5.51 7.85**	5.43 7.89
	PMB 0.5	5.00 6.74	4.96 7.00	5.02 6.74	5.02 7.23	6.17 9.07	5.36 6.99	5.32 8.11	5.30 7.73
	PMB 1	4.80 5.99	4.98 6.59	4.92 6.25	4.96 7.22	5.20 6.82	5.22 7.06	5.19 6.71	5.25 7.99
	PMB 2	4.78 6.37	4.78 7.04	4.79 7.01	4.88 6.79	5.19 7.80	5.16 6.51	5.18 6.22	5.29 7.73
**ARU622**	PMB 0	6.08 9.01	6.15 9.01	6.23 8.97	5.80 7.77	6.10 9.08	6.08 9.08	6.21 9.06	6.19 8.45
	PMB 0.25	6.09 9.03	6.14 9.07	6.21 8.97	5.07 5.99	6.03 9.08	5.94 9.02	6.20 8.94	**5.73 7.87**
	PMB 0.5	5.07 6.18	6.13 8.98	5.33 6.55	5.08 6.28	5.90 9.04	6.15 9.00	4.87 7.32	4.90 8.17
	PMB 1	5.37 7.16	4.48 5.92	4.85 5.83	4.80 5.96	5.90 8.81	5.31 8.13	4.86 7.22	4.84 8.56
	PMB 2	4.35 5.56	4.76 5.74	4.34 5.49	4.76 5.66	4.74 8.34	4.87 7.54	4.70 7.62	4.90 7.98
**ARU623**	PMB 0	6.02 9.05	6.27 8.94	6.11 8.96	5.18 7.81	6.06 9.03	6.22 8.98	6.33 8.95	5.28 8.53
	PMB 0.25	6.16 8.95	6.28 8.95	**4.82 7.28**	4.92 6.95	6.20 8.98	6.14 9.03	**5.01 6.93**	5.07 7.01
	PMB 0.5	5.96 8.95	4.61 8.09	4.79 8.22	4.77 8.24	6.10 8.81	4.46 7.05	4.89 6.50	4.98 6.78
	PMB 1	4.33 6.04	4.51 6.52	4.57 6.19	4.69 8.37	6.10 8.74	4.78 6.29	4.81 5.89	4.82 7.34
	PMB 2	4.00 6.14	3.92 8.39	3.79 7.36	4.40 7.34	4.02 6.78	4.36 6.18	4.13 6.05	4.47 7.34

Supplementary data.

Minocycline		MIN 0	MIN 0.5	MIN 4	MIN 16	MIN 0	MIN 0.5	MIN 4	MIN 16
**ARU617**	PMB 0	6.17 9.02	6.23 9.02	6.25 9.01	6.24 8.91	6.19 9.02	6.27 9.04	6.25 9.04	6.29 8.98
	PMB 0.25	6.22 9.02	6.24 9.02	6.22 9.02	6.20 8.84	6.19 9.04	6.25 9.04	6.28 9.03	**4.39 7.36**
	PMB 0.5	4.03 5.06	4.66 6.62	4.34 6.14	4.36 6.83	4.74 6.78	4.42 6.65	4.57 7.00	4.63 7.08
	PMB 1	4.34 5.55	4.06 6.04	4.23 5.93	4.27 6.28	3.93 6.39	4.25 6.44	4.30 7.01	4.78 7.53
	PMB 2	4.17 5.90	4.10 5.73	3.81 5.40	4.13 6.03	4.41 7.20	4.34 6.10	4.49 6.19	4.32 7.90
**ARU620**	PMB 0	5.91 9.07	6.06 9.07	6.11 9.07	6.16 8.83	5.99 9.02	6.07 9.01	6.17 8.99	6.15 8.48
	PMB 0.25	5.95 9.07	6.04 9.06	6.05 9.07	**5.15 6.73**	6.05 9.02	6.08 9.01	5.99 9.07	**5.51 7.28**
	PMB 0.5	5.00 6.74	5.15 6.90	5.09 6.90	5.07 6.75	6.17 9.07	5.46 6.93	5.44 6.90	5.48 7.29
	PMB 1	4.80 5.99	4.95 6.50	5.02 6.45	4.86 6.60	5.20 6.82	5.36 7.28	5.33 7.27	5.40 7.42
	PMB 2	4.78 6.37	4.95 6.47	4.92 6.12	4.84 6.59	5.19 7.80	5.28 7.88	5.25 7.32	5.21 7.99
**ARU622**	PMB 0	6.08 9.01	6.10 9.01	6.19 9.01	6.22 8.97	6.09 9.07	6.11 9.07	6.15 9.07	6.12 9.07
	PMB 0.25	6.09 9.03	6.08 9.02	6.22 9.00	6.16 8.96	6.11 9.07	6.11 9.07	6.14 9.07	**4.90 6.84**
	PMB 0.5	5.07 6.18	6.29 8.99	6.30 8.68	5.09 6.13	4.79 6.64	4.89 6.66	4.88 6.48	4.71 6.29
	PMB 1	5.37 7.16	5.04 6.52	4.97 6.09	4.88 6.14	4.57 6.05	4.21 6.76	4.61 5.93	4.34 5.66
	PMB 2	4.35 5.56	4.62 6.08	4.59 5.62	4.75 5.94	4.23 7.02	4.31 6.57	4.29 5.56	4.44 6.33
**ARU623**	PMB 0	6.02 9.05	6.26 8.93	6.25 8.94	6.12 8.91	6.06 9.03	6.24 8.97	6.28 8.98	6.18 8.71
	PMB 0.25	6.16 8.95	6.27 8.93	6.31 8.94	**4.94 7.46**	6.20 8.98	6.00 9.03	6.26 8.98	**5.08 6.62**
	PMB 0.5	5.96 8.95	4.97 6.51	4.93 7.10	4.88 6.93	6.10 8.81	4.74 6.80	5.04 6.41	5.09 6.54
	PMB 1	4.33 6.04	4.63 6.90	4.68 7.73	4.74 7.06	6.10 8.74	4.52 7.08	4.83 6.41	4.76 7.48
	PMB 2	4.00 6.14	4.44 6.57	5.10 7.59	4.50 7.62	4.02 6.78	4.39 6.92	4.26 6.14	4.09 7.57
**Temocillin**		TMC 0	TMC 4	TMC 16	TMC 64	TMC 0	TMC 4	TMC 16	TMC 64
**ARU617**	PMB 0	6.11 9.07	6.24 9.07	6.23 9.07	6.19 9.07	6.19 9.05	6.23 9.03	6.25 9.03	6.25 9.02
	PMB 0.25	6.20 9.04	6.18 9.05	6.17 9.06	6.06 9.01	6.27 9.03	6.27 9.02	6.24 9.03	6.28 9.02
	PMB 0.5	6.27 8.92	6.22 8.62	6.24 8.87	6.11 8.76	6.02 8.54	6.31 8.71	6.33 8.88	6.28 8.87
	PMB 1	3.96 8.24	4.47 8.63	6.22 8.65	4.18 7.43	5.04 7.91	4.92 6.67	4.77 6.42	4.62 6.97
	PMB 2	4.06 8.57	4.21 8.70	4.11 8.59	4.24 7.64	4.66 6.90	4.71 7.71	4.77 7.62	4.63 7.47
**ARU620**	PMB 0	6.02 8.99	6.24 8.99	6.18 9.01	6.13 9.01	6.07 9.07	6.10 9.01	6.13 9.02	6.09 9.03
	PMB 0.25	6.20 8.98	6.14 9.00	6.08 9.02	6.22 8.98	6.08 9.02	6.09 9.06	6.02 9.07	5.98 9.07
	PMB 0.5	5.90 8.77	6.16 8.75	6.10 8.83	**5.05 6.65**	5.10 8.65	5.99 8.98	5.11 6.59	5.07 6.40
	PMB 1	4.50 7.76	3.97 5.63	4.67 5.95	4.18 5.61	4.85 7.41	4.86 6.67	4.98 6.35	5.04 6.26
	PMB 2	4.33 8.09	3.95 4.98	4.22 7.89	4.69 7.78	4.70 8.44	4.67 8.68	4.51 8.64	4.55 8.58
**ARU622**	PMB 0	5.87 9.08	6.09 9.02	6.12 9.02	6.06 9.08	5.81 9.03	6.11 9.03	6.10 9.03	6.09 9.04
	PMB 0.25	6.03 9.03	6.07 9.07	6.08 9.07	6.09 9.01	6.10 9.04	6.07 9.07	6.10 9.03	6.10 9.03
	PMB 0.5	6.03 9.01	6.10 9.00	6.10 9.01	6.05 9.00	5.90 9.00	5.94 8.98	6.07 9.05	**5.24 8.58**
	PMB 1	4.78 7.79	6.09 8.19	4.96 6.84	5.98 8.91	5.80 8.76	5.63 8.70	6.03 8.77	6.02 8.89
	PMB 2	4.92 6.76	5.32 6.38	5.62 7.63	5.44 7.51	4.72 8.42	4.72 8.71	4.67 8.12	5.04 8.52
**ARU623**	PMB 0	6.02 8.99	6.24 8.99	6.18 9.01	6.13 9.01	6.18 8.99	6.18 9.00	6.15 9.01	6.05 9.02
	PMB 0.25	6.20 8.98	6.14 9.00	6.08 9.02	6.22 8.98	5.99 9.02	6.09 9.04	6.01 9.00	6.11 9.01
	PMB 0.5	5.90 8.77	6.16 8.75	6.10 8.83	**5.05 6.65**	4.97 7.52	6.02 8.85	4.81 8.70	4.91 7.13
	PMB 1	4.50 7.76	3.97 5.63	4.67 5.95	4.18 5.61	4.58 6.72	4.55 7.41	5.12 8.76	4.62 8.63
	PMB 2	4.33 8.09	3.95 4.98	4.22 7.89	4.69 7.78	4.48 8.31	4.31 8.78	4.33 8.75	4.90 8.65

Supplementary data.

**Thiamphenicol**		THI 0		THI 2		THI 8		THI 32		THI 0		THI 2		THI 8		THI 32	
	PMB 0	6.20	9.06	6.23	9.06	6.23	9.06	6.24	9.07	6.25	9.02	6.13	9.06	6.27	9.01	6.28	8.96
	PMB 0.25	6.19	9.02	6.19	9.01	6.25	9.00	6.24	9.04	6.22	9.06	6.21	9.00	6.28	8.98	6.27	8.92
**ARU617**	PMB 0.5	4.81	6.11	6.29	8.87	6.31	8.94	5.70	7.81	6.28	8.47	6.19	8.65	4.27	5.84	**4.63**	**6.02**
	PMB 1	4.47	6.44	4.43	6.57	4.20	6.10	4.56	6.42	4.36	6.50	5.85	7.70	4.59	5.88	3.81	6.09
	PMB 2	4.28	6.09	4.52	6.68	4.12	5.81	4.09	5.90	4.32	7.28	4.63	6.43	4.41	6.33	4.12	6.24
	PMB 0	6.02	8.99	6.24	8.98	6.16	9.05	6.07	8.97	6.07	9.07	6.09	9.01	6.14	8.99	6.22	8.97
	PMB 0.25	6.20	8.98	6.12	9.03	6.20	8.95	6.24	8.95	6.08	9.02	5.98	9.01	6.13	8.99	6.25	8.95
**ARU620**	PMB 0.5	5.90	8.77	4.77	5.87	4.87	7.02	**4.96**	**6.58**	5.10	8.65	6.01	9.07	5.21	7.64	5.14	8.79
	PMB 1	4.50	7.76	5.79	8.13	4.59	6.88	4.45	7.30	4.85	7.41	5.00	6.42	5.03	6.43	5.05	6.72
	PMB 2	4.33	8.09	4.53	7.53	4.83	7.18	4.72	7.28	4.70	8.44	4.64	8.46	4.77	8.12	4.60	8.11
	PMB 0	5.93	9.03	6.15	9.01	6.15	9.01	6.13	8.98	5.81	9.03	6.12	9.03	6.08	9.03	6.11	9.01
	PMB 0.25	6.10	9.02	5.90	9.07	6.09	9.07	6.04	9.06	6.10	9.04	6.07	9.03	6.09	9.07	6.04	9.00
**ARU622**	PMB 0.5	6.12	9.01	6.09	8.98	5.87	9.00	6.06	8.97	5.90	9.00	6.06	8.90	5.65	8.06	**5.34**	**7.64**
	PMB 1	4.96	6.37	6.07	8.36	5.02	6.58	5.19	6.89	5.80	8.76	5.80	8.01	5.02	7.32	5.05	6.90
	PMB 2	4.80	6.10	4.70	6.22	4.83	6.59	4.96	6.66	4.72	8.42	4.85	7.62	4.83	8.04	4.99	7.37
	PMB 0	6.02	8.99	6.24	8.98	6.16	9.05	6.07	8.97	6.18	8.99	6.12	9.02	5.99	9.05	6.08	9.04
	PMB 0.25	6.20	8.98	6.12	9.03	6.20	8.95	6.24	8.95	5.99	9.02	6.10	9.04	6.09	9.05	6.13	8.97
**ARU623**	PMB 0.5	5.90	8.77	4.77	5.87	4.87	7.02	**4.96**	**6.58**	4.97	7.52	4.69	6.66	4.93	6.84	4.84	7.64
	PMB 1	4.50	7.76	5.79	8.13	4.59	6.88	4.45	7.30	4.58	6.72	4.65	8.09	4.67	7.57	4.79	7.81
	PMB 2	4.33	8.09	4.53	7.53	4.83	7.18	4.72	7.28	4.48	8.31	4.55	8.57	4.69	7.68	4.43	7.66

**Trimethoprim**		TMP 0		TMP 1		TMP 4		TMP 8		TMP 0		TMP 1		TMP 4		TMP 8	
	PMB 0	6.20	9.06	6.20	9.06	6.23	9.06	6.09	9.06	6.21	9.07	6.25	9.06	6.25	9.03	6.26	9.03
	PMB 0.25	6.19	9.02	6.20	9.02	6.13	9.01	6.18	9.06	6.23	9.05	6.21	9.01	6.22	9.04	6.01	9.05
**ARU617**	PMB 0.5	4.81	6.11	6.32	8.92	6.31	8.91	6.31	8.75	6.24	8.83	4.09	6.21	**4.00**	**6.51**	6.28	8.74
	PMB 1	4.47	6.44	4.59	6.35	6.27	8.75	4.48	6.50	4.17	8.66	4.46	6.59	4.03	7.96	3.68	7.76
	PMB 2	4.28	6.09	4.37	6.30	4.20	6.11	4.26	6.42	4.60	7.75	4.44	8.66	4.19	8.63	4.13	8.59
	PMB 0	6.02	8.99	6.13	9.01	6.15	9.01	6.16	9.01	6.07	9.07	6.09	9.07	6.08	9.07	6.10	9.07
	PMB 0.25	6.20	8.98	6.24	8.98	6.26	8.98	6.11	8.99	6.08	9.02	6.08	9.01	6.07	9.01	6.10	9.01
**ARU620**	PMB 0.5	5.90	8.77	4.94	6.60	4.95	6.18	**4.78**	**6.38**	5.10	8.65	5.21	6.40	5.19	6.38	5.17	7.05
	PMB 1	4.50	7.76	4.70	7.16	4.21	5.86	4.70	7.65	4.85	7.41	5.03	7.35	5.07	7.65	5.01	8.40
	PMB 2	4.33	8.09	4.50	7.35	4.71	7.49	4.60	8.06	4.70	8.44	4.79	7.85	4.62	8.33	4.50	8.48
	PMB 0	5.81	9.03	6.13	9.07	6.12	9.07	6.04	9.05	5.97	9.08	5.82	9.04	6.00	9.08	5.94	9.08
	PMB 0.25	6.10	9.04	6.11	9.03	6.11	9.03	6.00	9.03	5.99	9.04	6.02	9.07	6.10	9.07	6.12	9.07
**ARU622**	PMB 0.5	5.90	9.00	6.04	8.84	5.86	7.89	**5.13**	**7.85**	5.91	9.07	6.08	8.93	5.00	8.40	**4.57**	**8.42**
	PMB 1	5.80	8.76	5.97	8.56	4.95	6.72	4.91	8.24	5.95	9.05	6.02	8.72	6.13	8.96	4.92	7.64
	PMB 2	4.72	8.42	4.72	7.51	5.07	7.03	4.68	8.11	4.17	8.77	4.80	8.72	4.82	8.71	4.71	8.67
	PMB 0	6.02	8.99	6.13	9.01	6.15	9.01	6.16	9.01	6.18	8.99	6.20	8.99	6.13	9.02	6.11	9.01
	PMB 0.25	6.20	8.98	6.24	8.98	6.26	8.98	6.11	8.99	5.99	9.02	6.11	9.02	6.11	9.03	6.06	9.04
**ARU623**	PMB 0.5	5.90	8.77	4.94	6.60	4.95	6.18	**4.78**	**6.38**	4.97	7.52	4.85	7.45	6.27	8.56	4.92	7.76
	PMB 1	4.50	7.76	4.70	7.16	4.21	5.86	4.70	7.65	4.58	6.72	4.67	7.99	4.76	7.96	4.73	7.03
	PMB 2	4.33	8.09	4.50	7.35	4.71	7.49	4.60	8.06	4.48	8.31	4.02	7.92	4.89	8.04	4.50	8.49

Supplementary data.

Rifampicin		RIF 0	RIF 1	RIF 8	RIF 32	RIF 0	RIF 1	RIF 8	RIF 32
**ARU617**	PMB 0	6.17 9.02	6.20 9.02	6.27 8.98	4.42 4.20	6.19 9.02	6.27 9.05	6.28 8.88	5.31 5.04
	PMB 0.25	6.22 9.02	6.23 9.01	6.29 8.97	4.95 5.59	6.19 9.04	6.23 9.05	6.30 8.87	4.66 5.79
	PMB 0.5	4.03 5.06	4.62 6.64	4.54 5.84	4.12 5.62	4.74 6.78	4.39 6.32	4.06 5.26	4.27 4.93
	PMB 1	4.34 5.55	3.80 6.16	4.01 5.40	4.14 5.65	3.93 6.39	4.01 6.28	4.38 6.03	4.12 5.58
	PMB 2	4.17 5.90	3.75 5.72	4.29 5.82	3.74 4.96	4.41 7.20	4.04 6.32	4.74 6.15	4.07 4.98
**ARU620**	PMB 0	5.91 9.07	6.05 9.07	6.01 9.06	5.29 5.38	5.99 9.02	6.07 9.02	6.12 9.00	5.28 5.58
	PMB 0.25	5.95 9.07	6.01 9.07	5.98 9.07	5.01 5.49	6.05 9.02	6.05 9.07	6.02 9.07	5.52 5.80
	PMB 0.5	5.00 6.74	5.09 6.80	5.05 6.09	4.93 5.06	6.17 9.07	5.40 6.55	**5.41 6.06**	5.23 6.01
	PMB 1	4.80 5.99	4.85 6.08	4.94 5.98	4.82 5.64	5.20 6.82	5.30 6.98	5.33 6.39	5.27 5.87
	PMB 2	4.78 6.37	4.80 6.06	4.52 5.51	4.67 5.32	5.19 7.80	5.28 7.41	5.10 5.67	4.97 5.69
**ARU622**	PMB 0	6.08 9.01	6.09 9.02	6.18 9.01	5.54 5.80	6.09 9.07	6.02 9.07	6.10 9.04	5.42 5.36
	PMB 0.25	6.09 9.03	6.11 9.07	6.25 8.97	5.09 6.63	6.11 9.07	6.05 9.07	6.13 9.05	4.99 5.94
	PMB 0.5	5.07 6.18	5.11 6.44	5.12 6.03	4.94 5.63	4.79 6.64	4.82 6.80	4.86 6.05	4.34 5.82
	PMB 1	5.37 7.16	4.96 6.23	4.82 6.04	4.79 5.74	4.57 6.05	4.52 6.40	4.13 5.78	4.46 5.88
	PMB 2	4.35 5.56	4.33 5.54	4.36 5.50	4.33 5.33	4.23 7.02	4.30 6.58	4.13 5.39	4.24 5.76
**ARU623**	PMB 0	6.02 9.05	6.18 8.94	6.23 8.75	4.70 5.30	6.06 9.03	6.25 8.97	6.23 8.86	5.43 5.64
	PMB 0.25	6.16 8.95	6.25 8.92	**5.73 7.52**	4.79 5.89	6.20 8.98	6.24 8.97	**5.61 7.14**	5.19 5.73
	PMB 0.5	5.96 8.95	4.86 6.87	4.81 5.70	4.52 5.32	6.10 8.81	5.10 6.25	5.15 5.83	4.94 5.33
	PMB 1	4.33 6.04	4.41 6.57	4.55 5.69	4.51 5.56	6.10 8.74	4.69 6.64	5.02 5.53	4.82 5.50
	PMB 2	4.00 6.14	4.46 6.59	4.16 5.55	4.11 5.62	4.02 6.78	4.44 6.80	4.17 5.10	4.67 5.62

Abbreviations: AMK, amikacin; ATM, aztreonam; FEP, cefepime; CHL, chloramphenicol; CIP, ciprofloxacin; FOF, fosfomycin; LIN, linezolid; MEM meropenem; MIN, minocycline; PMB, polymyxin B; RIF, rifampicin; TMC, temocillin; THI, thiamphenicol; TMP, trimethoprim.

Supplementary data

Table S2. Results of static time-kill experiments. Mean bacterial concentrations in log_{10} CFU/mL (standard deviations) at 0, 3, 6 and 24 hours, change in bacterial concentrations (Δ) compared to the starting inoculum, and combination interactions (Int), i.e. difference in bacterial concentrations with the combination compared to the most active single antibiotic, are presented. Synergy is highlighted in dark grey, additive effects in light grey and a bactericidal activity is marked in bold.

Strain	Antibiotics	0h	3h	$Δ_{3h}$	Int_{3h}	6h	$Δ_{6h}$	Int_{6h}	24h	$Δ_{24h}$	Int_{24h}
ARU617	PMB 0.5	6.55 (0.16)	2.04 (0.00)	**-4.13**		4.51 (0.36)	-1.66		7.97 (0.96)	1.80	
	ATM 64	6.44 (0.16)	5.50 (0.24)	-0.67		6.56 (0.04)	0.39		8.69 (0.08)	2.52	
	PMB 0.5 + ATM 64	6.46 (0.23)	1.00 (0.00)	**-6.17**	-2.04	1.69 (1.19)	**-4.48**	-2.82	1.50 (0.00)	**-6.17**	-7.97
	Control	6.47 (0.22)	7.98 (0.08)	1.81		8.91 (0.02)	2.74		9.70 (0.07)	3.53	
	PMB 0.5	6.55 (0.16)	4.08 (0.00)	-2.09		4.51 (0.36)	-1.66		7.97 (0.96)	1.80	
	FEP 64	6.52 (0.23)	5.07 (1.18)	-1.10		4.37 (0.18)	-1.80		5.21 (0.50)	-0.96	
	PMB 0.5 + FEP 64	6.54 (0.18)	3.85 (0.59)	-2.32	-0.23	2.89 (1.42)	**-3.28**	-1.48	2.69 (0.62)	**-3.48**	-2.52
	Control	6.47 (0.22)	7.98 (0.08)	1.81		8.91 (0.02)	2.74		9.70 (0.07)	3.53	
	PMB 0.5	7.01 (0.04)	1.59 (0.11)	**-4.58**		2.37 (0.04)	**-3.80**		8.90 (0.18)	2.73	
	FOF 128	6.55 (0.24)	4.42 (0.08)	-1.75		3.67 (0.34)	-2.50		9.55 (0.04)	3.38	
	PMB 0.5 + FOF 128	6.44 (0.14)	2.41 (0.71)	**-3.76**	0.82	2.07 (1.57)	**-4.10**	-0.30	3.26 (1.05)	-2.91	-5.64
	Control	6.47 (0.22)	7.98 (0.08)	1.81		8.91 (0.02)	2.74		9.70 (0.07)	3.53	
	PMB 0.25	6.50 (0.02)	6.60 (0.04)	0.43		7.94 (0.00)	1.77		9.67 (0.12)	3.50	
	MIN 16	6.53 (0.03)	6.64 (0.11)	0.47		5.98 (0.31)	-0.19		7.41 (0.75)	1.24	
	PMB 0.25 + MIN 16	6.53 (0.02)	1.24 (0.24)	**-4.93**	-5.36	1.00 (0.00)	**-5.17**	-4.98	2.71 (1.71)	**-3.46**	-4.70
	Control	6.52 (0.02)	7.97 (0.05)	1.80		8.77 (0.01)	2.60		9.69 (0.19)	3.52	
	PMB 0.5	6.42 (0.16)	7.36 (0.22)	1.19		8.52 (0.09)	2.35		9.70 (0.09)	3.53	
	THI 32	6.47 (0.24)	7.89 (0.01)	1.72		8.30 (0.29)	2.13		8.78 (0.13)	2.61	
	PMB 0.5 + THI 32	6.23 (0.07)	6.53 (0.31)	0.36	-0.83	7.60 (0.36)	1.43	-0.70	8.52 (0.16)	2.35	-0.26
	Control	6.38 (0.18)	7.80 (0.08)	1.63		8.70 (0.05)	2.53		9.59 (0.07)	3.42	
	PMB 0.5	6.42 (0.16)	7.36 (0.22)	1.19		8.52 (0.09)	2.35		9.70 (0.09)	3.53	
	TMP 4	6.47 (0.21)	7.85 (0.22)	1.68		8.37 (0.32)	2.20		9.75 (0.01)	3.58	
	PMB 0.5 + TMP 4	6.37 (0.13)	6.71 (0.31)	0.54	-0.65	8.01 (0.23)	1.84	-0.36	9.70 (0.08)	3.53	0.00
	Control	6.38 (0.18)	7.80 (0.08)	1.63		8.70 (0.05)	2.53		9.59 (0.07)	3.42	
ARU620	PMB 0.25	6.17 (0.00)	5.91 (0.64)	-0.26		7.33 (0.15)	1.16		9.47 (0.17)	3.30	
	AMK 4	6.22 (0.09)	6.64 (0.90)	0.47		3.76 (0.00)	-2.41		9.08 (0.21)	2.91	
	PMB 0.25 + AMK 4	6.09 (0.08)	3.21 (0.00)	-2.96	-2.70	3.69 (0.00)	-2.48	-0.07	8.13 (1.05)	1.96	-0.95
	Control	6.15 (0.06)	7.50 (0.04)	1.33		8.55 (0.15)	2.38		9.46 (0.07)	3.29	
	PMB 0.25	5.75 (0.04)	3.17 (1.27)	**-3.00**		4.14 (1.39)	-2.03		9.17 (0.15)	3.00	
	ATM 64	5.83 (0.05)	3.72 (0.76)	**-2.45**		3.00 (0.00)	**-3.17**		5.13 (0.42)	-1.04	
	PMB 0.25 + ATM 64	5.87 (0.00)	1.50 (0.50)	**-5.67**	-2.67	1.00 (0.00)	**-6.17**	-3.00	1.20 (1.70)	**-4.97**	-3.93
	Control	5.85 (0.05)	7.64 (0.04)	1.47		8.63 (0.06)	2.46		9.72 (0.03)	3.55	
	PMB 0.25	5.75 (0.04)	3.17 (1.27)	**-3.00**		4.14 (1.39)	-2.03		9.17 (0.15)	3.00	
	FEP 64	5.83 (0.10)	1.43 (1.43)	**-4.74**		3.45 (0.05)	-2.72		5.17 (0.50)	-1.00	
	PMB 0.25 + FEP 64	5.76 (0.06)	1.00 (0.00)	**-6.17**	-1.43	1.00 (0.00)	**-6.17**	-3.45	1.39 (0.89)	**-4.78**	-3.78
	Control	5.85 (0.05)	7.64 (0.04)	1.47		8.63 (0.06)	2.46		9.72 (0.03)	3.55	
	PMB 0.5	6.01 (0.02)	2.52 (1.22)	**-3.65**		1.71 (0.71)	**-4.46**		6.93 (2.10)	0.76	
	CHL 32	6.06 (0.01)	7.28 (0.11)	1.11		7.82 (0.03)	1.65		9.06 (0.23)	2.89	
	PMB 0.5 + CHL 32	6.08 (0.09)	1.00 (0.00)	**-5.17**	-1.52	1.00 (0.00)	**-5.17**	-0.71	1.00 (0.00)	**-5.17**	-5.93
	Control	6.11 (0.01)	7.67 (0.09)	1.50		8.47 (0.07)	2.30		9.52 (0.01)	3.35	
	PMB 0.25	5.75 (0.04)	3.17 (1.27)	**-3.00**		4.14 (1.39)	-2.03		9.17 (0.15)	3.00	
	FOF 128	5.86 (0.11)	5.12 (0.16)	-1.05		5.18 (0.22)	-0.99		9.30 (0.02)	3.13	
	PMB 0.25 + FOF 128	5.77 (0.07)	3.28 (0.28)	-2.89	0.11	2.72 (0.65)	**-3.45**	-1.42	9.15 (0.20)	2.98	-0.01
	Control	5.85 (0.05)	7.64 (0.04)	1.47		8.63 (0.06)	2.46		9.72 (0.03)	3.55	
	PMB 0.5	5.88 (0.05)	5.15 (0.95)	-1.02		5.98 (1.78)	-0.19		9.26 (0.44)	3.09	
	LIN 8	5.97 (0.15)	7.49 (0.05)	1.32		8.56 (0.02)	2.39		9.59 (0.09)	3.42	
	PMB 0.5 + LIN 8	6.06 (0.12)	5.45 (1.00)	-0.72	0.30	6.60 (1.40)	0.43	0.62	9.53 (0.14)	3.36	0.27
	Control	5.97 (0.13)	7.56 (0.22)	1.39		8.53 (0.12)	2.36		9.67 (0.05)	3.50	

Supplementary data

Strain	Antibiotics	0h	3h	Δ_{3h}	Int_{3h}	6h	Δ_{6h}	Int_{6h}	24h	Δ_{24h}	Int_{24h}
	PMB 0.25	6.17 (0.00)	5.91 (0.64)	-0.26		7.33 (0.15)	1.16		9.47 (0.17)	3.30	
	MEM 16	6.18 (0.02)	1.88 (1.38)	**-4.29**		3.28 (0.24)	-2.89		5.48 (0.02)	-0.69	
	PMB 0.25 + MEM 16	6.22 (0.02)	1.74 (0.26)	**-4.43**	-0.14	1.50 (0.00)	**-5.67**	-2.78	3.18 (0.00)	-2.99	-2.29
	Control	6.15 (0.06)	7.50 (0.04)	1.33		8.55 (0.15)	2.38		9.46 (0.07)	3.29	
	PMB 0.25	6.17 (0.00)	5.91 (0.64)	-0.26		7.33 (0.15)	1.16		9.47 (0.17)	3.30	
	MIN 16	6.19 (0.12)	6.93 (0.26)	0.76		7.86 (0.24)	1.69		8.97 (0.03)	2.80	
	PMB 0.25 + MIN 16	6.18 (0.02)	3.34 (1.34)	-2.83	-2.57	3.59 (1.55)	-2.58	-3.74	8.35 (0.00)	2.18	-0.62
	Control	6.15 (0.06)	7.50 (0.04)	1.33		8.55 (0.15)	2.38		9.46 (0.07)	3.29	
	PMB 0.5	6.01 (0.02)	2.52 (1.22)	**-3.65**		1.71 (0.71)	**-4.46**		6.93 (2.10)	0.76	
	RIF 8	6.12 (0.01)	7.47 (0.00)	1.30		8.11 (0.15)	1.94		8.72 (0.11)	2.55	
	PMB 0.5 + RIF 8	6.00 (0.10)	1.00 (0.00)	**-5.17**	-1.52	1.00 (0.00)	**-5.17**	-0.71	1.00 (0.00)	**-5.17**	-5.93
ARU620	Control	6.11 (0.01)	7.67 (0.09)	1.50		8.47 (0.07)	2.30		9.52 (0.01)	3.35	
	PMB 0.5	5.88 (0.05)	5.15 (0.95)	-1.02		5.98 (1.78)	-0.19		9.26 (0.44)	3.09	
	TMC 64	6.00 (0.00)	7.27 (0.18)	1.10		8.00 (0.03)	1.83		8.98 (0.05)	2.81	
	PMB 0.5 + TMC 64	6.11 (0.10)	4.39 (0.04)	-1.78	-0.76	5.92 (0.80)	-0.25	-0.06	8.72 (0.25)	2.55	-0.27
	Control	5.97 (0.13)	7.56 (0.22)	1.39		8.53 (0.12)	2.36		9.67 (0.05)	3.50	
	PMB 0.5	5.88 (0.05)	5.15 (0.95)	-1.02		5.98 (1.78)	-0.19		9.26 (0.44)	3.09	
	THI 32	6.15 (0.16)	7.35 (0.09)	1.18		8.14 (0.26)	1.97		8.87 (0.09)	2.70	
	PMB 0.5 + THI 32	6.04 (0.11)	3.95 (1.54)	-2.22	-1.20	5.23 (1.67)	-0.94	-0.75	7.06 (2.01)	0.89	-1.81
	Control	5.97 (0.13)	7.56 (0.22)	1.39		8.53 (0.12)	2.36		9.67 (0.05)	3.50	
	PMB 0.5	5.88 (0.05)	5.15 (0.95)	-1.02		5.98 (1.78)	-0.19		9.26 (0.44)	3.09	
	TMP 8	5.94 (0.11)	7.51 (0.19)	1.34		8.58 (0.13)	2.41		9.53 (0.06)	3.36	
	PMB 0.5 + TMP 8	5.92 (0.20)	5.00 (1.49)	-1.17	-0.15	6.42 (1.52)	0.25	0.44	9.40 (0.10)	3.23	0.14
	Control	5.97 (0.13)	7.56 (0.22)	1.39		8.53 (0.12)	2.36		9.67 (0.05)	3.50	
	PMB 0.25	6.36 (0.01)	7.58 (0.00)	1.41		8.57 (0.04)	2.40		9.83 (0.20)	3.66	
	ATM 64	6.34 (0.04)	5.37 (0.10)	-0.80		5.13 (0.62)	-1.04		6.02 (0.07)	-0.15	
	PMB 0.25 + ATM 64	6.27 (0.10)	4.37 (0.07)	-1.80	-1.00	4.26 (0.22)	-1.91	-0.87	5.64 (0.17)	-0.53	-0.38
	Control	6.37 (0.10)	7.98 (0.09)	1.81		8.68 (0.03)	2.51		9.92 (0.18)	3.75	
	PMB 0.5	6.32 (0.13)	2.26 (0.96)	**-3.91**		3.22 (0.48)	-2.95		8.81 (0.38)	2.64	
	FEP 8	6.29 (0.17)	3.69 (0.00)	-2.48		4.27 (0.24)	-1.90		5.67 (0.13)	-0.50	
	PMB 0.5 + FEP 8	6.38 (0.11)	3.49 (1.08)	-2.68	1.23	1.96 (0.96)	**-4.21**	-1.26	3.24 (0.01)	-2.93	-2.43
	Control	6.30 (0.01)	7.97 (0.05)	1.80		8.58 (0.05)	2.41		9.61 (0.09)	3.44	
	PMB 0.5	6.28 (0.09)	3.42 (2.12)	-2.75		3.38 (0.64)	-2.79		8.88 (0.45)	2.71	
	FOF 128	6.31 (0.16)	5.56 (0.32)	-0.61		5.38 (0.07)	-0.79		9.49 (0.01)	3.32	
	PMB 0.5 + FOF 128	6.43 (0.30)	2.37 (0.29)	**-3.80**	-1.05	1.00 (0.00)	**-5.17**	-2.38	1.00 (0.00)	**-5.17**	-7.88
	Control	6.39 (0.08)	8.02 (0.00)	1.85		8.71 (0.09)	2.54		9.72 (0.02)	3.55	
	PMB 0.5	6.42 (0.05)	2.57 (0.29)	**-3.60**		3.32 (0.14)	-2.85		8.99 (0.51)	2.82	
	LIN 16	6.42 (0.05)	7.92 (0.09)	1.75		8.53 (0.00)	2.36		9.59 (0.14)	3.42	
	PMB 0.5 + LIN 16	6.44 (0.05)	4.00 (0.60)	-2.17	1.43	4.73 (0.50)	-1.44	1.41	8.54 (1.05)	2.37	-0.45
	Control	6.38 (0.07)	7.86 (0.02)	1.69		8.45 (0.02)	2.28		9.65 (0.15)	3.48	
	PMB 0.25	6.67 (0.37)	6.86 (0.89)	0.19		8.09 (0.26)	1.42		9.63 (0.09)	2.97	
ARU622	MEM 64	6.58 (0.10)	3.57 (0.14)	**-3.01**		3.20 (0.02)	**-3.38**		3.98 (0.88)	-0.65	
	PMB 0.25 + MEM 64	6.44 (0.12)	2.94 (1.47)	**-3.50**	-1.46	2.06 (1.06)	**-4.38**	-0.63	2.24 (1.24)	**-5.55**	-1.74
	Control	6.62 (0.00)	8.07 (0.00)	1.45		8.78 (0.00)	2.16		9.58 (0.00)	3.28	
	PMB 0.25	6.51 (0.00)	5.18 (1.13)	-0.99		5.76 (2.27)	-0.41		9.60 (0.15)	3.43	
	MIN 16	6.43 (0.01)	7.69 (0.12)	1.52		8.43 (0.02)	2.26		9.35 (0.21)	3.18	
	PMB 0.25 + MIN 16	6.45 (0.06)	5.87 (0.02)	-0.30	0.69	6.59 (0.08)	0.42	0.83	9.34 (0.12)	3.17	-0.01
	Control	6.44 (0.02)	8.09 (0.01)	1.92		8.57 (0.06)	2.40		9.79 (0.00)	3.62	
	PMB 0.5	6.36 (0.03)	7.70 (0.02)	1.53		8.46 (0.07)	2.29		9.68 (0.01)	3.51	
	TMC 64	6.39 (0.07)	7.80 (0.09)	1.63		8.23 (0.01)	2.06		9.61 (0.08)	3.44	
	PMB 0.5 + TMC 64	6.35 (0.00)	7.35 (0.02)	1.18	-0.35	8.17 (0.05)	2.00	-0.06	9.64 (0.13)	3.47	0.03
	Control	6.38 (0.07)	7.86 (0.02)	1.69		8.45 (0.02)	2.28		9.65 (0.15)	3.48	
	PMB 0.5	6.37 (0.09)	2.00 (0.08)	**-4.17**		2.98 (0.35)	**-3.19**		8.25 (0.99)	2.08	
	THI 32	6.48 (0.16)	7.62 (0.09)	1.45		8.44 (0.07)	2.27		9.03 (0.22)	2.86	
	PMB 0.5 + THI 32	6.38 (0.07)	2.48 (0.70)	**-3.69**	0.48	2.13 (0.23)	**-4.04**	-0.85	3.88 (1.38)	-2.29	-4.36
	Control	6.42 (0.05)	7.74 (0.19)	1.57		8.63 (0.00)	2.46		9.76 (0.09)	3.59	
	PMB 0.5	6.37 (0.09)	2.00 (0.08)	**-4.17**		2.98 (0.35)	**-3.19**		8.25 (0.99)	2.08	
	TMP 8	6.41 (0.06)	7.94 (0.12)	1.77		8.63 (0.06)	2.46		9.59 (0.03)	3.42	
	PMB 0.5 + TMP 8	6.39 (0.08)	2.70 (1.10)	**-3.47**	0.70	3.36 (0.12)	-2.81	0.38	8.69 (0.36)	2.52	0.44
	Control	6.42 (0.05)	7.74 (0.19)	1.57		8.63 (0.00)	2.46		9.76 (0.09)	3.59	

Supplementary data

Strain	Antibiotics	0h	3h	Δ_{3h}	Int_{3h}	6h	Δ_{6h}	Int_{6h}	24h	Δ_{24h}	Int_{24h}
ARU623	PMB 0.25	6.25 (0.04)	6.16 (0.06)	-0.01		7.60 (0.11)	1.43		9.72 (0.19)	3.55	
	AMK 16	6.34 (0.08)	1.00 (0.00)	**-6.17**		2.30 (0.02)	**-3.87**		8.58 (0.07)	2.41	
	PMB 0.25 + AMK 16	6.23 (0.03)	1.80 (0.10)	**-4.37**	1.80	1.39 (0.09)	**-4.78**	-0.91	7.63 (0.05)	1.46	-0.95
	Control	6.31 (0.04)	7.89 (0.03)	1.72		9.13 (0.01)	2.96		9.59 (0.18)	3.42	
	PMB 0.25	5.88 (0.26)	6.30 (0.86)	0.13		7.68 (0.77)	1.51		9.35 (0.23)	3.18	
	ATM 64	5.87 (0.26)	5.12 (0.49)	-1.05		5.12 (0.66)	-1.05		6.14 (0.18)	-0.03	
	PMB 0.25 + ATM 64	5.82 (0.22)	3.89 (0.17)	-2.28	-1.23	3.38 (0.49)	-2.79	-1.74	5.08 (0.37)	-1.09	-1.06
	Control	5.94 (0.29)	7.80 (0.22)	1.63		8.40 (0.19)	2.23		9.45 (0.08)	3.28	
	PMB 0.25	5.88 (0.26)	6.30 (0.86)	0.13		7.68 (0.77)	1.51		9.35 (0.23)	3.18	
	FEP 64	5.77 (0.24)	4.36 (0.00)	-1.81		4.13 (0.00)	-2.04		5.77 (0.04)	-0.40	
	PMB 0.25 + FEP 64	5.92 (0.12)	3.22 (0.32)	-2.95	-1.14	2.36 (0.10)	**-3.81**	-1.77	4.12 (0.08)	-2.05	-1.65
	Control	5.94 (0.29)	7.80 (0.22)	1.63		8.40 (0.19)	2.23		9.45 (0.08)	3.28	
	PMB 0.5	6.10 (0.12)	2.25 (0.32)	**-3.92**		1.95 (0.47)	**-4.22**		5.40 (1.04)	-0.77	
	CHL 32	6.09 (0.01)	6.98 (0.04)	0.81		7.79 (0.01)	1.62		8.89 (0.54)	2.72	
	PMB 0.5 + CHL 32	6.17 (0.05)	2.52 (0.16)	**-3.65**	0.27	2.80 (0.20)	**-3.37**	0.85	4.79 (0.45)	-1.38	-0.61
	Control	6.16 (0.05)	7.57 (0.06)	1.40		8.32 (0.07)	2.15		9.32 (0.22)	3.15	
	PMB 0.25	6.25 (0.04)	6.16 (0.06)	-0.01		7.60 (0.11)	1.43		9.72 (0.19)	3.55	
	CIP 2	6.28 (0.02)	4.76 (0.23)	-1.41		4.13 (0.39)	-2.04		7.67 (0.59)	1.50	
	PMB 0.25 + CIP 2	6.24 (0.09)	1.67 (0.67)	**-4.50**	-3.09	2.50 (0.72)	**-3.67**	-1.63	8.34 (0.23)	2.17	0.67
	Control	6.31 (0.04)	7.89 (0.03)	1.72		9.13 (0.01)	2.96		9.59 (0.18)	3.42	
	PMB 0.25	6.03 (0.19)	6.18 (0.37)	0.01		7.11 (0.57)	0.94		9.45 (0.01)	3.28	
	FOF 128	6.01 (0.18)	6.47 (0.57)	0.30		6.53 (0.90)	0.36		9.38 (0.15)	3.21	
	PMB 0.25 + FOF 128	5.96 (0.12)	4.56 (0.82)	-1.61	-1.62	4.94 (1.47)	-1.23	-1.59	9.08 (0.38)	2.91	-0.31
	Control	6.05 (0.18)	7.71 (0.00)	1.54		8.19 (0.17)	2.02		9.38 (0.15)	3.21	
	PMB 0.25	6.67 (0.37)	6.86 (0.88)	0.69		8.08 (0.26)	1.91		9.62 (0.09)	3.45	
	MEM 16	6.57 (0.09)	3.56 (0.13)	-2.61		3.19 (0.01)	-2.98		3.98 (0.87)	-2.19	
	PMB 0.25 + MEM 16	6.43 (0.11)	2.94 (1.46)	**-3.23**	-0.62	2.06 (1.06)	**-4.11**	-1.13	2.24 (1.24)	**-3.93**	-1.74
	Control	6.61 (0.00)	8.07 (0.00)	1.90		8.77 (0.00)	2.60		9.58 (0.00)	3.41	
	PMB 0.25	6.20 (0.08)	7.60 (0.01)	1.43		8.80 (0.00)	2.63		8.82 (0.76)	2.65	
	MIN 16	6.20 (0.07)	4.61 (0.00)	-1.56		4.58 (0.76)	-1.59		7.35 (0.31)	1.18	
	PMB 0.25 + MIN 16	6.16 (0.15)	1.81 (1.81)	**-4.36**	-2.80	1.65 (1.65)	**-4.52**	-2.93	3.60 (3.60)	-2.57	-3.75
	Control	6.08 (0.06)	7.74 (0.01)	1.57		8.54 (0.36)	2.37		9.02 (0.59)	2.85	
	PMB 0.25	6.13 (0.01)	7.67 (0.06)	1.50		8.78 (0.00)	2.61		8.88 (0.82)	2.71	
	RIF 8	6.16 (0.10)	6.38 (0.22)	0.21		7.40 (0.00)	1.23		6.66 (0.80)	0.49	
	PMB 0.25 + RIF 8	6.14 (0.19)	3.09 (3.09)	**-3.08**	-3.29	0.00 (2.91)	**-6.17**	-7.40	2.83 (2.33)	**-3.34**	-3.33
	Control	6.03 (0.02)	7.83 (0.08)	1.66		8.40 (0.22)	2.23		8.97 (0.54)	2.80	
	PMB 0.5	6.10 (0.12)	2.25 (0.32)	**-3.92**		1.95 (0.47)	**-4.22**		5.40 (1.04)	-0.77	
	TMC 64	6.07 (0.02)	7.47 (0.04)	1.30		7.96 (0.03)	1.79		9.25 (0.11)	3.08	
	PMB 0.5 + TMC 64	6.11 (0.04)	2.22 (0.91)	**-3.95**	-0.03	2.54 (1.06)	**-3.63**	0.59	6.81 (0.19)	0.64	1.41
	Control	6.16 (0.05)	7.57 (0.06)	1.40		8.32 (0.07)	2.15		9.32 (0.22)	3.15	
	PMB 0.5	5.92 (0.06)	6.62 (0.10)	0.45		8.20 (0.03)	2.03		8.63 (0.29)	2.46	
	THI 32	6.02 (0.05)	6.82 (0.01)	0.65		8.08 (0.06)	1.91		8.00 (0.10)	1.83	
	PMB 0.5 + THI 32	5.92 (0.07)	5.27 (0.64)	-0.90	-1.35	6.64 (1.64)	0.47	-1.44	8.72 (0.71)	2.55	0.72
	Control	5.92 (0.16)	7.29 (0.04)	1.12		8.15 (0.03)	1.98		8.83 (0.35)	2.66	
	PMB 0.5	6.10 (0.12)	2.25 (0.32)	**-3.92**		1.95 (0.47)	**-4.22**		5.40 (1.04)	-0.77	
	TMP 8	6.08 (0.09)	7.52 (0.03)	1.35		8.38 (0.10)	2.21		9.36 (0.04)	3.19	
	PMB 0.5 + TMP 8	6.10 (0.04)	1.74 (0.26)	**-4.43**	-0.51	1.15 (0.15)	**-5.02**	-0.80	3.95 (0.46)	-2.22	-1.45
	Control	6.16 (0.05)	7.57 (0.06)	1.40		8.32 (0.07)	2.15		9.32 (0.22)	3.15	

Abbreviations: AMK, amikacin; ATM, aztreonam; FEP, cefepime; CHL, chloramphenicol; CIP, ciprofloxacin; FOF, fosfomycin; LIN, linezolid; MEM meropenem; MIN, minocycline; PMB, polymyxin B; RIF, rifampicin; TMC, temocillin; THI, thiamphenicol; TMP, trimethoprim.
